# Associations between infection intensity categories and morbidity prevalence in school-age children are much stronger for *Schistosoma haematobium* than for *S. mansoni*

**DOI:** 10.1371/journal.pntd.0009444

**Published:** 2021-05-25

**Authors:** Ryan E. Wiegand, W. Evan Secor, Fiona M. Fleming, Michael D. French, Charles H. King, Arminder K. Deol, Susan P. Montgomery, Darin Evans, Jürg Utzinger, Penelope Vounatsou, Sake J. de Vlas

**Affiliations:** 1 Division of Parasitic Diseases and Malaria, Centers for Disease Control and Prevention, Atlanta, Georgia, United States of America; 2 Swiss Tropical and Public Health Institute, Basel, Switzerland; 3 University of Basel, Basel, Switzerland; 4 SCI Foundation, London, United Kingdom; 5 RTI International, Washington DC, United States of America; 6 Center for Global Health and Diseases, Case Western Reserve University, Cleveland, Ohio, United States of America; 7 Department of Infectious Disease Epidemiology, London School of Hygiene and Tropical Medicine, London, United Kingdom; 8 United States Agency for International Development, Washington DC, United States of America; 9 Department of Public Health, Erasmus MC, University Medical Center Rotterdam, Rotterdam, The Netherlands; Wellcome Sanger Institute, UNITED KINGDOM

## Abstract

**Background:**

World Health Organization (WHO) guidelines for measuring global progress in schistosomiasis control classify individuals with *Schistosoma* spp. infections based on the concentration of excreted eggs. We assessed the associations between WHO infection intensity categories and morbidity prevalence for selected *S*. *haematobium* and *S*. *mansoni* morbidities in school-age children.

**Methodology:**

A total of 22,488 children aged 6–15 years from monitoring and evaluation cohorts in Burkina Faso, Mali, Niger, Uganda, Tanzania, and Zambia from 2003–2008 were analyzed using Bayesian logistic regression. Models were utilized to evaluate associations between intensity categories and the prevalence of any urinary bladder lesion, any upper urinary tract lesion, microhematuria, and pain while urinating (for *S*. *haematobium*) and irregular hepatic ultrasound image pattern (C-F), enlarged portal vein, laboratory-confirmed diarrhea, and self-reported diarrhea (for *S*. *mansoni*) across participants with infection and morbidity data.

**Principal findings:**

*S*. *haematobium* infection intensity categories possessed consistent morbidity prevalence across surveys for multiple morbidities and participants with light infections had elevated morbidity levels, compared to negative participants. Conversely, *S*. *mansoni* infection intensity categories lacked association with prevalence of the morbidity measures assessed.

**Conclusions/significance:**

Current status infection intensity categories for *S*. *haematobium* were associated with morbidity levels in school-age children, suggesting urogenital schistosomiasis morbidity can be predicted by an individual’s intensity category. Conversely, *S*. *mansoni* infection intensity categories were not consistently indicative of childhood morbidity at baseline or during the first two years of a preventive chemotherapy control program.

## Author summary

Infections with *Schistosoma* parasites are commonly classified by the presence and concentration of excreted *Schistosoma* eggs. Guidelines put forward by the World Health Organization (WHO) include classifications of *S*. *haematobium* infections assessed by urine filtration into light and heavy infections and *S*. *mansoni* infections assessed by Kato-Katz thick smears into light, moderate, and heavy infections. Past evidence has demonstrated an association between intensity of infection with morbidity for severe morbidities, but this was before recognition of the effect of light-intensity infections on morbidity and was done in treatment naïve populations. In these analyses, we assessed the associations between the WHO classifications for infection intensity and a wide array of *S*. *haematobium* and *S*. *mansoni* morbidity indicators in school-age children ascertained in monitoring and evaluation cohorts before and after initiation of deworming. Our analyses found a high correlation with *S*. *haematobium* intensity categories and morbidity indicators, especially microhematuria, but weaker correlation between *S*. *mansoni* intensity categories and morbidity indicators. The results indicate that, on the aggregate, the intensity categories represent a person’s *S*. *haematobium*-related morbidity but are poor at representing a person’s *S*. *mansoni*-related morbidity.

## Introduction

Schistosomiasis is the disease caused by infection with the blood fluke *Schistosoma* spp. Morbidity is caused by the host’s response to parasite eggs. As part of the life cycle, eggs are excreted via urine (for *Schistosoma haematobium*) or stool (for *S*. *mansoni* and other species), but some eggs become lodged in host tissue and stimulate inflammation and granulomatous reactions, which are responsible for the pathology associated with the infection [1]. Chronic disease can manifest due to the accumulation of tissue damage by repeated infections or because schistosomes can survive in the human body and produce eggs for many years. Morbidity varies by species [1,2]. *S*. *haematobium* infections affect the urogenital system and most often clinically present as hematuria [3]. Chronic infections can result in urinary tract fibrosis [4], female [5] and male [6] genital schistosomiasis, and, in rare cases, bladder cancer [7]. *S*. *mansoni* infections affect the gastrointestinal tract and frequently are associated with abdominal pain and bleeding into the stool [3]. Longer term infections put patients at greater risk for periportal fibrosis [1], which can lead to portal vein hypertension, hepatosplenic disease, and esophageal varices that can result in exsanguination into the digestive tract.

Prior to the introduction of mass distribution of praziquantel as preventive chemotherapy, multiple studies in the 1970s and early 1980s found an association between the intensity of a schistosome infection and morbidity [8,9]. These studies became the basis for two important components of schistosomiasis morbidity control. First, they established the concept of infection intensity categorizations, now commonly used by the World Health Organization (WHO). *S*. *haematobium* infection intensity has consistently been characterized by the number of schistosome eggs per 10 ml of urine with 1–49 eggs per 10 ml of urine defining a light infection and ≥50 eggs per 10 ml of urine indicating a heavy infection [10,11]. For *S*. *mansoni*, infection intensity is measured as the number of schistosome eggs per gram (EPG) of stool. Different categorizations have appeared in WHO documents [10,12], but infection intensity is now commonly split into three categories: 1–99 EPG for a light infection; 100–399 EPG signifying a moderate infection; and ≥400 EPG for a heavy infection [11,13]. Second, they led to current WHO guidelines that use community-level prevalence of heavy-intensity infections as the basis for morbidity control [14], even though it is recognized that light and moderate infections can cause considerable morbidity [2,15].

The primary objective of this study was to determine whether the intensity categories, i.e., a measure of someone’s current infection status, can differentiate between participant’s morbidity prevalence for multiple, schistosomiasis-related morbidity indicators. For *S*. *haematobium*, we analyzed two aggregated ultrasound indicators, as well as microhematuria, and pain while urinating. For *S*. *mansoni*, we analyzed irregular liver image pattern, enlarged portal vein, laboratory-confirmed diarrhea, and self-reported diarrhea. We used data from preventive chemotherapy control programs in Burkina Faso [16–18], Mali [18,19], Niger [18], Uganda [20], Tanzania [21], and Zambia [21] between 2003 and 2008 supported by the Schistosomiasis Control Initiative (SCI) [22]. To our knowledge, no thorough evaluation of the association between infection intensity categories and morbidity before and after the mass distribution of praziquantel has been performed.

## Methods

### Ethics statement

The Imperial College Research Ethics Committee (ICREC_8_2_2, EC No. 03.36, R&D No. 03/SB/003E) and the ethical review boards of the Ministries of Health of the six countries included here provided ethical approval for use of these data. The Centers for Disease Control and Prevention (CDC) was determined to be a non-engaged research partner.

### Study design and data collection

Data collection was performed as part of national control programs for schistosomiasis and soil-transmitted helminthiasis. Details on country programs and development of SCI are presented elsewhere [16–22]. Briefly, all countries created a multifaceted, national program scale-up and initiated distribution of praziquantel and albendazole to target populations based on WHO guidance [23]. Schools were randomly selected from areas with various endemicity levels that were purposively selected. Countries created two monitoring and evaluation cohorts: a randomly selected group of children, aged 6–12 years in primary education, were followed for two or more years; and a second cohort of communities in the same geographic areas were tracked with a random sample of participants ascertained each year. The first survey (baseline) was the first known year of preventive chemotherapy in the area. The subsequent years of follow-up are referred to as follow up 1 and follow up 2. Data were collected from communities which had been treated annually and measured approximately one year after treatment. Treatment was usually immediately following survey times, but occasionally lagged by a few months. Details on surveys included in the analyses and reasons for exclusions are included in the supplementary materials (Table A in S1 Text). Participants were required to be above 94 cm in height and not currently ill. Consent was obtained from parents or guardians and assent was obtained from children. Participants were surveyed and evaluated before receiving praziquantel (for schistosomiasis) and albendazole (for soil-transmitted helminthiasis). We pooled the monitoring and evaluation cohorts and limited the participants to 6-15-year-old children to include the largest number of participants possible and to align with the current monitoring and evaluation guidelines of sampling school-age children [24]. Datasets were created for each morbidity where participants were required to possess both infection and morbidity data.

### *Schistosoma* infection data

Each control program chose independently how to evaluate *S*. *haematobium* and *S*. *mansoni* infections (Table 1). Urine filtration was used to determine *S*. *haematobium* infection intensity. A single urine sample was used in Burkina Faso, Niger, Tanzania, and Zambia. All countries performed a single filtration except Niger that prepared two filtrations from one urine sample. In Mali, program officials took two urine samples from consecutive days and filtered each. A stained filter was microscopically examined for *S*. *haematobium* eggs after passing approximately 10 ml of urine through it. An individual’s infection intensity was calculated as the arithmetic mean of the number of eggs per 10 ml of urine across all available samples. Participants were then grouped into three intensity infection categories: negative (0 eggs per 10 ml urine), light (1–49 eggs per 10 ml of urine), and heavy (≥50 eggs per 10 ml of urine) [11].

**Table 1:** Summary of *Schistosoma* infection data collection procedures by country, Schistosomiasis Control Initiative (SCI) supported monitoring and evaluation data, 2003–2008.

Country	*S*. *haematobium* 10 ml urine filtrations	*S*. *mansoni* 41.7mg Kato-Katz (KK) slides
Burkina Faso	One	One stool sample with two slides
Mali	Two from different samples taken on consecutive days	One stool sample with two slides
Niger	Two from the same urine sample	One stool sample with two slides
Tanzania	One	Two stool samples with two slides
Uganda	No evaluation	Baseline: one stool sample with two slides Follow-up: two stool samples with two slides
Zambia	One	Two stool samples with two slides

*S*. *mansoni* infection intensity was determined by the Kato-Katz technique. Mali, Niger, and the baseline Ugandan survey used a single stool with counting of two separately prepared Kato-Katz thick smears. Two stools from consecutive days with two slides each were used in Tanzania and all follow-up surveys in Uganda. Slides were microscopically examined for *S*. *mansoni* eggs. Individual intensity of infection was determined by calculating the arithmetic mean of the number of eggs on all available slides multiplied by a factor of 24 to scale the measurement to eggs per 1 g of stool. Participants were classified into four intensity infection categories: negative (0 EPG), light (1–99 EPG), moderate (100–399 EPG), and heavy (≥400 EPG) [11].

### Morbidity data

Morbidity data were collected from the same participants as the infection data. *S*. *haematobium* ultrasound variables utilized in these analyses included all elements of an *S*. *haematobium* ultrasound evaluation [25] performed in accordance to the standardized Niamey protocol [26]. These included a distorted bladder shape, the presence of irregularities in the bladder wall, detection of any bladder wall masses >10 mm in size, presence of pseudopolyps, a focal or diffuse thickening of the bladder wall, any dilation of the left or right pelvis, and any visualization of the left or right ureter. The first five indicators were aggregated into an outcome denoting whether a participant was positive for any urinary bladder lesion while the pelvic dilation and ureter visualization indicators were aggregated into an outcome denoting whether a participant was positive for any upper urinary tract lesion. Participants missing any of the *S*. *haematobium* ultrasound indicators were excluded from these analyses. Additionally, as a sensitivity analysis, a second approach utilized the number of positive indicators and the total number of indicators measured and are referred to as the urinary bladder rate and upper urinary tract rate. All participants with at least one indicator measured were included in the second analysis. Two additional morbidity indicators were also analyzed: microhematuria assessed with Hemastix dipsticks [27] and self-reported pain while urinating [28]. The analyses of individual morbidity indicators are included in the supplementary material (S1 Text).

Four main morbidity variables were analyzed for *S*. *mansoni*. Two ultrasound indicators of *S*. *mansoni* infection, collected according to the Niamey protocol [26], were included in analyses: the presence of a hepatic image pattern of C or worse to measure where a participant has any echogenic fibrosis; and an enlarged portal vein, defined as a portal vein diameter with a score of >2 standard deviations from the Senegalese population data used in the Niamey protocol. Unfortunately, other indicators were not consistently measured in all countries. The final two morbidity variables for *S*. *mansoni* were laboratory-confirmed and self-reported diarrhea [29,30]. Laboratory-confirmed diarrhea was indicated by the technician, while self-reported diarrhea was based on participants’ responses when asked if they had experienced diarrhea in the last 2 weeks. Analyses of hepatic irregular image pattern B or worse, blood in stool, and abdominal pain for *S*. *mansoni* are included in the supplementary materials (S1 Text).

### Data analysis methods

R version 4.0.3 [31] and the tidyverse package [32] were used to prepare data for analyses. All analyses that required a level of significance used the 5% level. Analyses included in this report’s figures treated the data as a stratified (country level) and clustered (school level) sample via the survey package [33]. Logistic regression was used to compare the relative odds of possessing a morbidity between categories within a survey. These models have the goal of comparing participants’ current infection status within the same survey and do not make comparisons between surveys or identify the best infection categories.

The regression model contained fixed effects for countries to control for differences between control programs, age and sex of participants, survey year, intensity infection category, and the interaction between survey year and intensity category. A random intercept for each school was included to account for any correlation between children sampled from the same school. Participants who contributed data at multiple surveys had individual random intercepts, while those participants who contributed for a single year did not. All comparisons were estimated via contrast statements and reported as odds ratios (ORs) with 95% Bayesian credible intervals (BCIs) from the posterior distributions. Regression models were fit via Markov chain Monte Carlo (MCMC) methods in JAGS [34] using the rjags package [35]. Three chains were chosen with an adaptive phase of 10,000 iterations and a total of 80,000 iterations per chain. The first 10,000 iterations were discarded. All fixed effect coefficients used have Cauchy prior distributions with a center of zero and a scale of 2.5 [36]. For *S*. *haematobium*-related morbidities, additional models were run without controlling for participants’ age and sex for comparison.

In addition, we explored whether using three categories of *S*. *mansoni* infection intensity (rather than four levels) might show a stronger association with morbidity indicator prevalence. Multiple categorizations were used to define the new *S*. *mansoni* infection intensity categories, each with zero EPG of stool treated as negative and the remaining two categories were split at a new threshold. We chose thresholds of 100 EPG, 200 EPG, 300 EPG, and 400 EPG to split infected participants. Thus, for example, the threshold of 200 EPG, produced a categorization of 0 EPG, 1–199 EPG, and ≥200 EPG.

## Results

### *S*. *haematobium*

**Fig 1:**
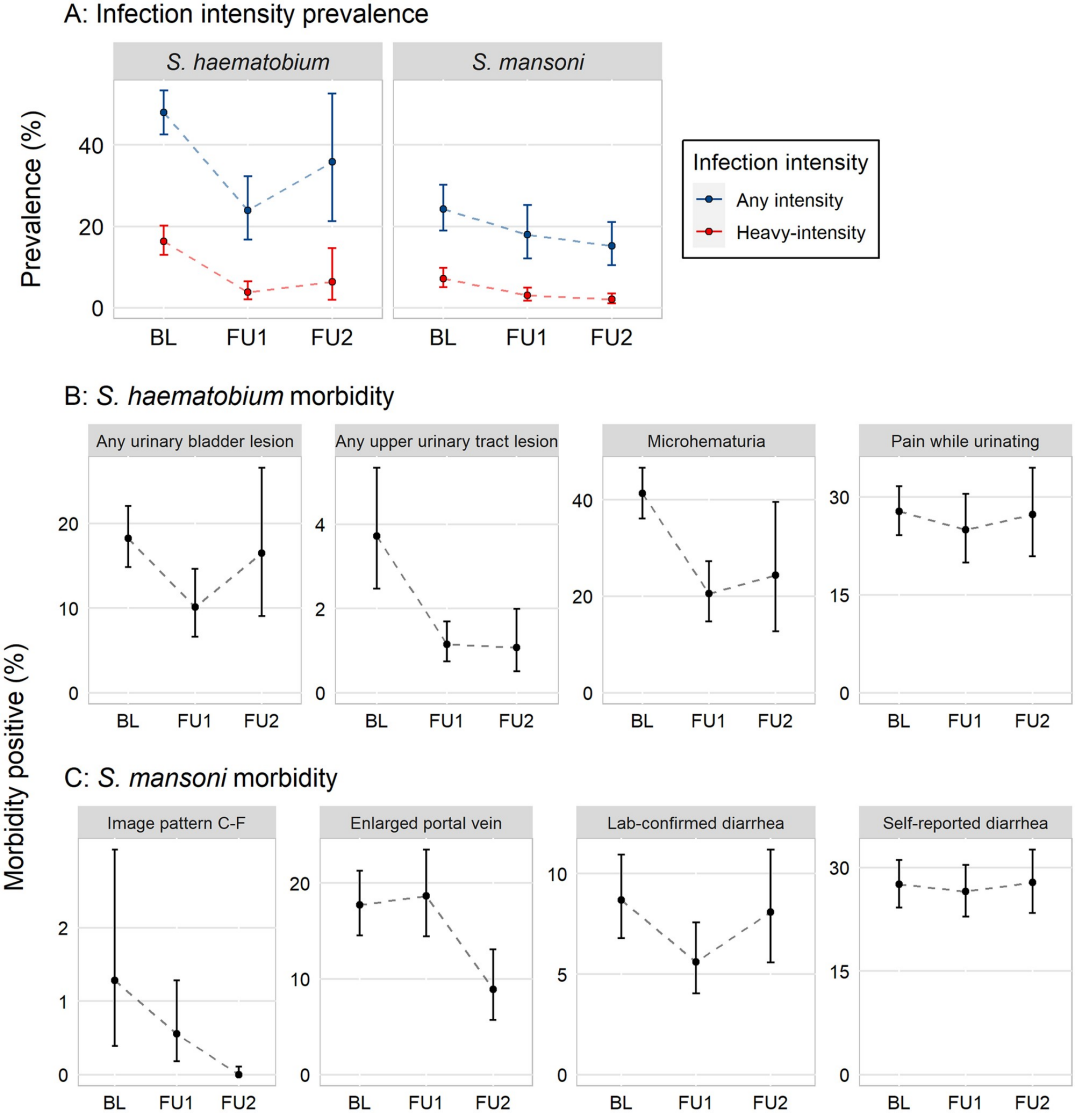
Line graphs of the percentage of 6- to 15-year-old children who were *Schistosoma* infection and heavy-intensity positive (row A), *S*. *haematobium* morbidity positive (row B), and *S*. *mansoni* morbidity positive (row C) at each survey year (baseline, BL; follow-up 1, FU1; follow-up 2, FU2). *S*. *haematobium* estimates are from Mali, Niger, and Tanzania and are assessed by urine filtration. *S*. *mansoni* estimates are from Mali, Niger, Tanzania, and Uganda and are assessed by the Kato-Katz technique.

A range of 8,615 to 11,948 school-age children were included at baseline depending on each morbidity’s missing data (Table B in S1 Text). This dropped to a range of 5,107 to 6,941 for follow-up 1 and 3,599 to 3,672 for follow-up 2. The prevalence of any infection and heavy-intensity infection prevalence were demonstrably higher for participants sampled at baseline compared to those sampled at follow-up 1 as the confidence intervals did not overlap; however, infection levels did not differ between follow-up 1 and follow-up 2 (Fig 1, row A). Similarly, most morbidities were more prevalent among participants sampled at baseline compared to participants sampled at follow-up 1 or at follow-up 2 (Fig 1, row B and A in S1 Text).

**Fig 2:**
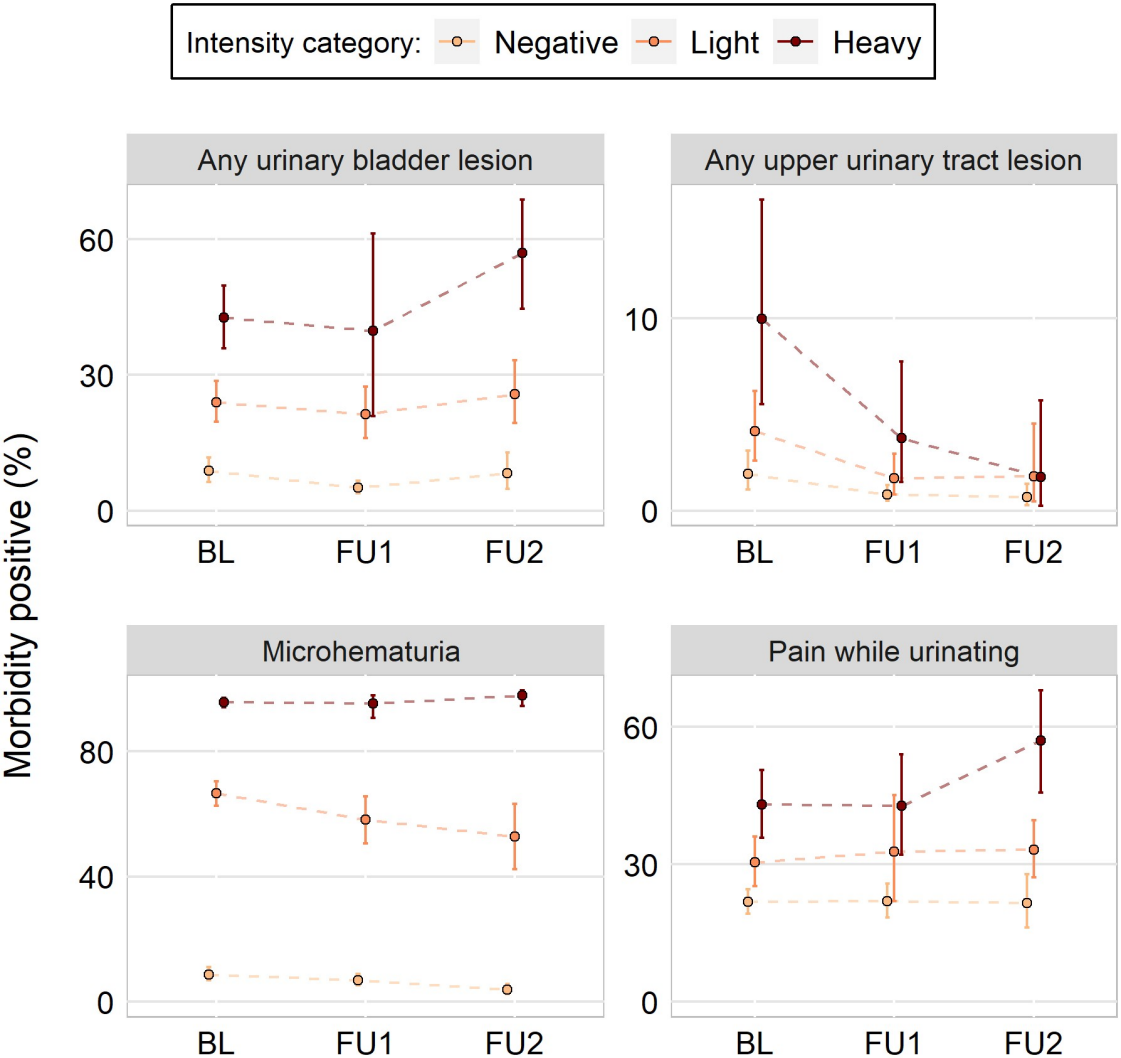
Line graphs of *Schistosoma haematobium*-related morbidity percentages, broken down by intensity category, across three surveys (baseline, BL; follow-up 1, FU1; follow-up 2, FU2). Participants were enrolled between 2003 and 2008 in Mali, Niger, and Tanzania. Clustering by school accounted for in 95% confidence bands. Infections were assessed by urine filtration.

With a few exceptions, infection intensity category prevalence estimates for *S*. *haematobium*-related morbidity indicators were consistent across surveys (Fig 2). Microhematuria prevalence was almost identical in each intensity category across surveys. Participants in the heavy intensity category at follow up 2 had a much higher prevalence of any urinary bladder lesion and the prevalence of pain while urinating; those morbidity indicators were consistent across surveys for the other intensity categories. The prevalence of any upper urinary tract lesion trended downward, for participants in the heavy intensity infection category, in each successive survey. Individual ultrasound indicators followed similar patterns but tended to have more variability (Table C in S1 Text).

ORs comparing participants in different intensity categories at each survey were roughly consistent for any urinary bladder lesions, any upper urinary tract lesions, microhematuria, and pain while urinating (Table 2). Compared to negative participants, participants with light infections were at increased odds for all morbidity indicators. The same was also true for participants with heavy infections, with the exception of the follow-up 2 survey for any upper urinary tract lesions. There was a lack of consistency between participants with heavy versus light infections for any upper urinary tract lesion, but differences were consistent at all timepoints for other morbidities. Similar results were found for binomial outcomes (Table C in S1 Text).

**Table 2:** Odds ratios (ORs) and 95% credible intervals from Bayesian logistic regression models comparing morbidity positive proportions between intensity categories within surveys for *S*. *haematobium*-related morbidities. Bold font indicates the 95% Bayesian credible interval (BCI) does not contain one. Participants are school-age children (6–15 years), enrolled between 2003 and2008 in Mali, Niger, and Tanzania.

Morbidity	Survey	Light versus negative	Heavy versus negative	Heavy versus light
Any urinary bladder lesions	Baseline	**1.42 (1.22, 1.64)**	**1.76 (1.46, 2.11)**	**1.24 (1.04, 1.48)**
	Follow-up1	**1.58 (1.33, 1.87)**	1.39 (0.99, 1.94)	0.88 (0.62, 1.23)
	Follow-up2	**2.32 (1.85, 2.90)**	**2.73 (1.93, 3.85)**	1.18 (0.84, 1.65)
Any upper urinary tract lesions	Baseline	**1.39 (1.02, 1.89)**	**1.74 (1.22, 2.48)**	1.26 (0.92, 1.72)
	Follow-up1	1.19 (0.81, 1.74)	1.67 (0.86, 3.02)	1.41 (0.72, 2.58)
	Follow-up2	1.96 (0.96, 4.02)	1.88 (0.54, 5.29)	0.96 (0.28, 2.67)
Microhematuria	Baseline	**2.11 (1.92, 2.33)**	**3.25 (2.88, 3.68)**	**1.54 (1.36, 1.73)**
	Follow-up1	**2.19 (1.91, 2.53)**	**2.84 (2.17, 3.74)**	1.29 (0.97, 1.73)
	Follow-up2	**2.00 (1.69, 2.38)**	**4.40 (3.27, 5.93)**	**2.19 (1.62, 2.98)**
Pain while urinating	Baseline	**1.19 (1.07, 1.34)**	**1.58 (1.38, 1.81)**	**1.32 (1.15, 1.52)**
	Follow-up1	1.02 (0.87, 1.20)	0.93 (0.67, 1.27)	0.91 (0.65, 1.27)
	Follow-up2	**1.27 (1.06, 1.54)**	1.26 (0.90, 1.77)	0.99 (0.70, 1.40)

The differences in the odds of possessing a morbidity between intensity categories was dramatically different when age and sex were not controlled for in regression models (Table 3). When age and sex are not included, the OR estimates are more closely aligned with those found in the raw data (Fig 2), than those in models with age and sex included (Table 2).

**Table 3:** Odds ratios (ORs) and 95% credible intervals from Bayesian logistic regression models comparing morbidity positive proportions between intensity categories within surveys for *S*. *haematobium*-related morbidities without controlling for age and sex. Bold font indicates the 95% Bayesian credible interval (BCI) does not contain one. Participants are school-age children (6–15 years), enrolled between 2003 and2008 in Mali, Niger, and Tanzania.

Morbidity	Survey	Light versus negative	Heavy versus negative	Heavy versus light
Any urinary bladder lesions	Baseline	**3.49 (2.98, 4.10)**	**10.15 (8.37, 12.34)**	**2.91 (2.46, 3.44)**
	Follow-up1	**3.89 (3.15, 4.80)**	**13.10 (9.36, 18.36)**	**3.37 (2.42, 4.70)**
	Follow-up2	**3.08 (2.47, 3.86)**	**10.07 (7.17, 14.23)**	**3.26 (2.34, 4.58)**
Any upper urinary tract lesions	Baseline	**1.75 (1.30, 2.35)**	**3.35 (2.43, 4.63)**	**1.92 (1.46, 2.51)**
	Follow-up1	1.15 (0.66, 1.94)	**2.77 (1.29, 5.46)**	**2.41 (1.08, 5.03)**
	Follow-up2	1.93 (0.99, 3.80)	1.63 (0.47, 4.43)	0.84 (0.25, 2.27)
Microhematuria	Baseline	**23.75 (20.67, 27.35)**	**296.27 (229.36, 388.21)**	**12.47 (9.90, 15.95)**
	Follow-up1	**14.45 (12.10, 17.29)**	**249.79 (143.44, 470.70)**	**17.28 (10.02, 32.37)**
	Follow-up2	**29.17 (22.56, 38.24)**	**1165.70 (506.17, 3363.16)**	**39.82 (17.86, 112.05)**
Pain while urinating	Baseline	**1.41 (1.26, 1.58)**	**2.40 (2.10, 2.75)**	**1.70 (1.48, 1.94)**
	Follow-up1	**1.59 (1.35, 1.86)**	**2.37 (1.77, 3.16)**	**1.49 (1.09, 2.03)**
	Follow-up2	**1.58 (1.31, 1.91)**	**4.58 (3.31, 6.38)**	**2.89 (2.07, 4.06)**

### *S*. *mansoni*

The sample sizes for *S*. *mansoni* analyses ranged from 5,158 to 13,483 school-age children at baseline, 4,881 to 11,484 for follow-up 1, and 3,901 to 6,216 for follow-up 2 (Table D in S1 Text). Although the prevalence of any infection and heavy-intensity infection prevalence was higher for participants sampled at baseline, as compared to those sampled at follow-up 1, the declines were modest and confidence intervals overlap (Fig 1, row A and C in S1 Text). *S*. *mansoni*-related morbidities, collected via ultrasound or in the laboratory and validated by a technician, also experienced decreases when participants at each follow-up survey are compared to baseline participants (Fig 1, row C and D in S1 Text), though the confidence intervals often overlap. The prevalence of enlarged portal vein slightly increased for participants in follow up 1, compared to baseline. Self-reported morbidities remained similar across the surveys.

**Fig 3:**
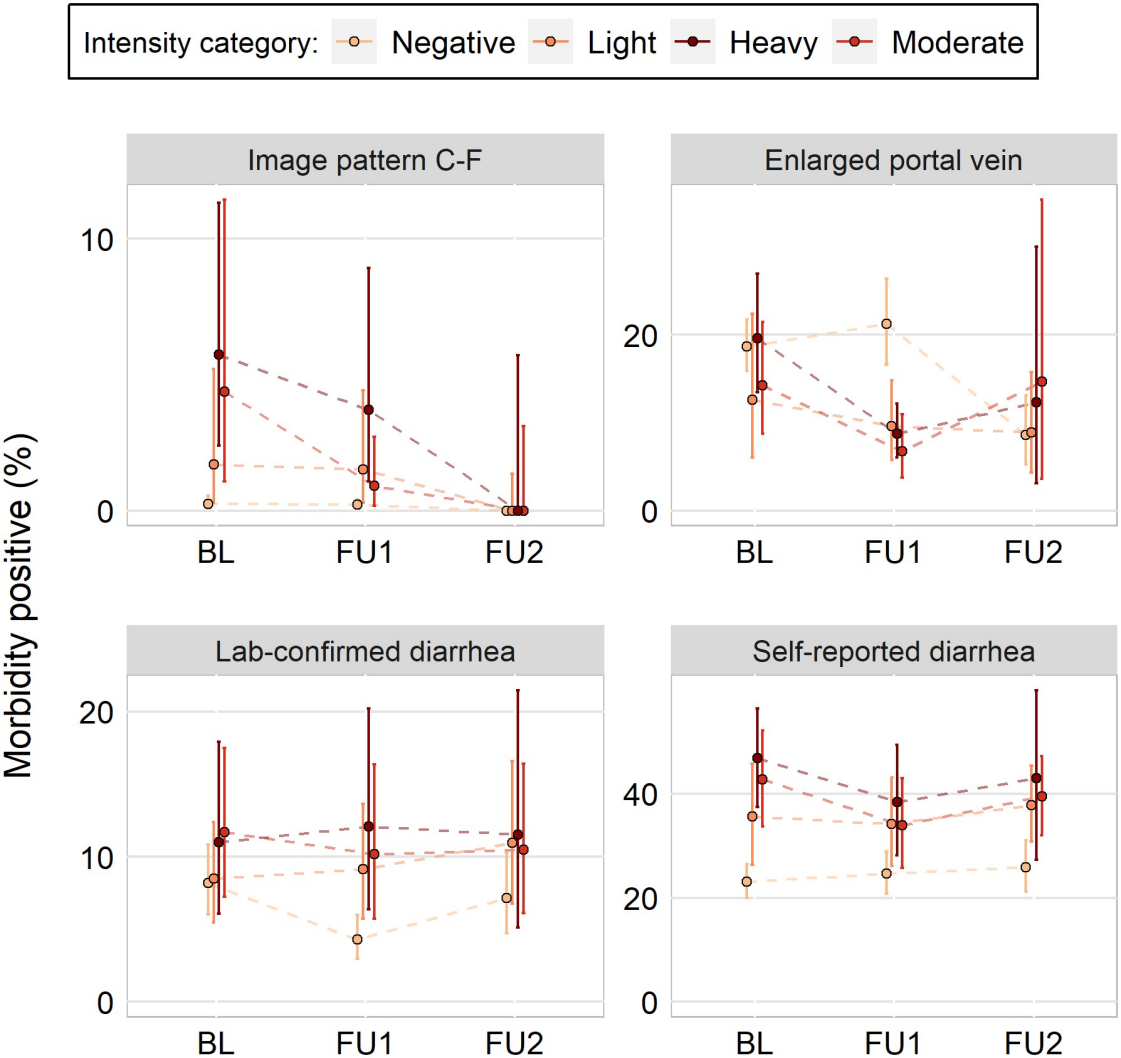
Line graphs of *Schistosoma mansoni*-related morbidity percentages, broken down by intensity category, across three surveys (baseline, BL; follow-up 1, FU1; follow-up 2, FU2). Participants were enrolled between 2003 and 2008 in Mali, Niger, Tanzania, and Uganda. Clustering by school accounted for in 95% confidence bands. Infections were assessed by Kato-Katz stool examination.

In contrast to *S*. *haematobium*, *S*. *mansoni*-related morbidities demonstrated no clear pattern and fewer associations with intensity categories (Fig 3; Table 4 and D in S1 Text). For instance, while baseline participants with heavy and moderate intensity infections were more likely to have an enlarged portal vein than their negative counterparts (moderate versus negative: OR = 1.53, 95% BCI = 1.09–2.13; heavy versus negative: OR = 2.48, 95% BCI = 1.86–3.30), these associations were much weaker at follow-up 1 (moderate versus negative: OR = 1.31, 95% BCI = 0.86–1.97; heavy versus negative: OR = 1.25, 95% BCI = 0.70–2.12), but then were much stronger at follow up 2 (moderate versus negative: OR = 3.29, 95% BCI = 1.91–5.47; heavy versus negative: OR = 4.68, 95% BCI = 2.24–9.18). Percentages of enlarged portal vein broken down by country (Fig E in S1 Text), show very high estimates of enlarged portal vein for Nigerien and Zambian participants with no infection. This resulted in negative participants having a higher estimate of enlarged portal vein than other intensities at both follow-up surveys (Fig 3). Though, since the modeled results take into account differences between countries and the age and sex of the participant, the ORs represent a more accurate comparison between the intensity categories (Table 4). Results for all *S*. *mansoni* morbidity indicators are included in the supplementary material (Table E in S1 Text).

**Table 4:** Odds ratios (ORs) and 95% credible intervals from Bayesian logistic regression models comparing morbidity positive proportions between heavy-intensity prevalence categories within surveys for *S*. *mansoni*-related morbidities. Bold font indicates the 95% Bayesian credible interval does not contain one. Participants are school-age children (6–15 years), enrolled between 2003 and2008 in Mali, Niger, Tanzania, and Uganda.

Morbidity	Survey	Light versus negative	Moderate versus negative	Heavy versus negative	Moderate versus light	Heavy versus light	Heavy versus moderate
Image pattern C-F	Baseline	1.95 (0.79, 5.05)	2.08 (0.86, 5.39)	**3.97 (1.67, 10.19)**	1.06 (0.50, 2.29)	2.03 (0.97, 4.37)	1.91 (0.96, 3.85)
	Follow-up 1	1.13 (0.45, 2.90)	0.70 (0.22, 2.12)	0.43 (0.07, 1.81)	0.62 (0.21, 1.68)	0.38 (0.06, 1.47)	0.62 (0.09, 2.69)
	Follow up 2	*	*	*	*	*	*
Enlarged portal vein	Baseline	0.98 (0.74, 1.27)	**1.53 (1.09, 2.13)**	**2.48 (1.86, 3.30)**	**1.57 (1.06, 2.31)**	**2.55 (1.79, 3.63)**	**1.62 (1.11, 2.39)**
	Follow-up 1	1.18 (0.83, 1.65)	1.31 (0.86, 1.97)	1.25 (0.70, 2.12)	1.11 (0.68, 1.79)	1.06 (0.57, 1.91)	0.95 (0.49, 1.79)
	Follow-up 2	1.34 (0.81, 2.12)	**3.29 (1.91, 5.47)**	**4.68 (2.24, 9.18)**	**2.46 (1.26, 4.82)**	**3.50 (1.52, 7.82)**	**1.42 (0.61, 3.23)**
Laboratory-confirmed diarrhea	Baseline	**1.32 (1.05, 1.66)**	**1.58 (1.23, 2.02)**	**1.69 (1.32, 2.15)**	1.20 (0.89, 1.60)	1.28 (0.96, 1.71)	1.07 (0.80, 1.44)
	Follow-up 1	**1.77 (1.38, 2.27)**	**1.76 (1.28, 2.40)**	**1.68 (1.14, 2.43)**	0.99 (0.70, 1.40)	0.95 (0.63, 1.42)	0.96 (0.61, 1.48)
	Follow-up 2	**1.47 (1.08, 1.97)**	1.32 (0.80, 2.11)	0.86 (0.42, 1.60)	0.90 (0.52, 1.51)	0.59 (0.28, 1.13)	0.65 (0.28, 1.41)
Self-reported diarrhea	Baseline	**1.17 (1.03, 1.33)**	**1.56 (1.34, 1.83)**	**1.43 (1.23, 1.66)**	**1.34 (1.11, 1.61)**	**1.22 (1.02, 1.46)**	0.91 (0.75, 1.11)
	Follow-up 1	1.14 (0.98, 1.33)	1.18 (0.96, 1.45)	1.29 (1.00, 1.66)	1.04 (0.82, 1.32)	1.13 (0.86, 1.49)	1.09 (0.80, 1.48)
	Follow-up 2	1.16 (0.97, 1.40)	1.29 (0.96, 1.73)	1.09 (0.76, 1.57)	1.11 (0.80, 1.54)	0.94 (0.64, 1.39)	0.85 (0.54, 1.33)

* Due to the low prevalence of image pattern C-F, all comparisons between categories were unstable and possessed a large amount of uncertainty; therefore they were omitted from this table.

**Fig 4:**
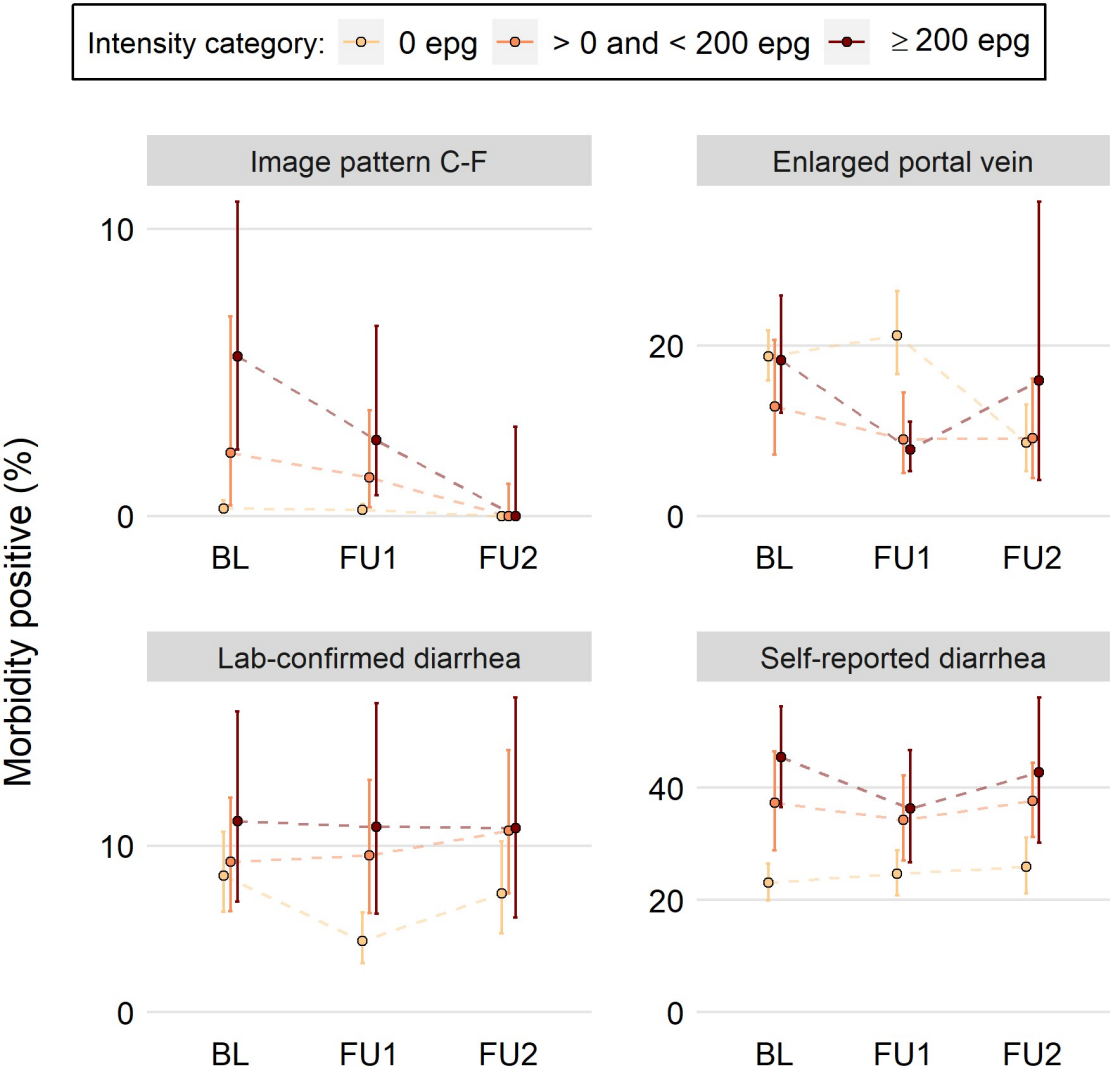
Modification of Fig 3 where participants’ *Schistosoma mansoni* intensity is split into three categories: 0 EPG, 1–199 EPG, and ≥200 EPG. See Fig 3 description for more details. Data are from three surveys (baseline, BL; follow-up 1, FU1; follow-up 2, FU2).

Under the consideration that the different patterns observed between *S*. *haematobium* and *S*. *mansoni* could be a statistical artifact of the number of intensity categories between species, we explored using three *S*. *mansoni* intensity categories instead of four. Using 200 EPG as the breakpoint between the light- and heavy-intensity categories, patterns similar to the four-category definition were seen (Fig 4). This was also true when 100 EPG, 300 EPG, and 400 EPG were utilized as the breakpoint between a light and heavy infection (Fig E-H in S1 Text).

## Discussion

These analyses indicated that there were consistent associations between infection intensity categories and the prevalence of some morbidity indicators for children aged 6–15 years for *S*. *haematobium*, as measured prior to the initiation of preventive chemotherapy (the baseline survey) and before each subsequent round of annual mass drug administration (follow-up 1 and follow-up 2 surveys). However, the same associations were not found for *S*. *mansoni* for the measures of morbidity that were collected. For the infection categories to effectively discriminate between morbidity directly associated with schistosomiasis, the odds of morbidity should be greater for a higher intensity category. ORs between *S*. *haematobium* infection intensity categories were largely consistent for most morbidity indicators before and after praziquantel administration. For example, across the three surveys, participants with light intensity infections had approximately two (or 15 to 30 without controlling for age and sex) times higher odds of microhematuria compared to participants without *S*. *haematobium* eggs in their urine. Consistent ORs were also found for heavy versus negative intensity. For *S*. *mansoni*, there was no morbidity measure where ORs between infection intensity categories were consistent. When raw prevalence and confidence intervals were plotted (Fig 3 and D in S1 Text), the categories are indistinguishable as confidence intervals overlap for almost all categories. This transpired in the context of effective preventive chemotherapy programs [37].

Our analyses had some limitations. Only morbidity indicators that were measured in multiple countries were analyzed, meaning many indicators from the *S*. *mansoni* ultrasound protocol were omitted. Some of those indicators suffer from high intra-observer variability [38–40], which suggest that those measures may not have been as useful as anticipated. Of note, enlarged portal vein measurements were taken against the Senegalese population utilized in the Niamey protocol, which may not be an appropriate comparison given these data come from other countries. The collection of stools (for Kato-Katz thick smears) and urine (for urine filtrations) varied across countries. Furthermore, additional stool and urine samples would have provided a more accurate assessment of infection and infection intensity. Although similar data systems were used by most countries, there was variability in the data collected, meaning that only subsets of the SCI-supported countries appear in most analyses. While recommendations were made for an appropriate number of schools and participants per school to enroll, data collection was a part of monitoring and evaluation cohorts without specific hypotheses. In addition, control programs had the final say on sample sizes and utilized different approaches, which were likely driven by available financial and human resources in some countries. While we realize these sample size considerations influenced the analyses, we were limited in our ability to power these analyses appropriately given the context in which the data were collected. Finally, there is the potential for systematic selection bias in these analyses as the school-age children sampled from these communities may be consistently different from those who were not sampled. In addition, while some people were measured multiple times, others were not, meaning the loss to follow up is high in these data. Nevertheless, if the intensity categories, which are purported to measure a person’s current infection status, have a robust relationship with morbidity, then we should see a similar relationship regardless of whether selection bias, heterogeneous age distributions, and other potential biases are present.

Nevertheless, an important finding is that participants with light and moderate *S*. *haematobium* infections had elevated morbidity levels compared to their non-infected counterparts for ultrasound indicators and microhematuria. This appears to be at odds with the notion that morbidity is only caused by heavy-infections [41], which has driven monitoring and evaluation goals for schistosomiasis [11,14,42]. Our results add to the literature that morbidity is present in people with even light and moderate infections [2,43] and strengthens the evidence-base that morbidity control should be based on any infection instead of heavy infections [15].

We explored some potential reasons for the stronger association between *S*. *haematobium* infection categories and morbidity, as compared to the *S*. *mansoni* infection categories and morbidity. One consideration was whether the poorer performance could be due to *S*. *mansoni* possessing a fourth intensity category compared to three for *S*. *haematobium*. Additional analyses were performed to evaluate whether separating *S*. *mansoni* intensity into three categories would improve discrimination. None of the multiple thresholds chosen hinted at an improvement to the association between infection intensity categories and morbidity.

Differences between species could be due to the diagnostic tests. Both the urine filtration and Kato-Katz techniques have considerable day-to-day variability [44,45]. Separate studies of the day-to-day fluctuation in these tests using the intra-person correlation coefficient found reasonably similar estimates for *S*. *haematobium* in Gabon (0.81, 95% confidence interval (CI): 0.71–0.89) [46] and *S*. *mansoni* in Burundi (0.77, 95% CI: 0.66–0.85) [47]. Though, the latter result differed from a study in Côte d’Ivoire where the intra-specimen variation of egg counts was 4.3 times higher than the day-to-day variability [45]. A comparable analysis could not be found for *S*. *mansoni* egg counts, but this variation could explain the worse performance of the Kato-Katz technique. Finally, small changes in *S*. *mansoni* egg counts have a much larger effect on the final intensity measure than for *S*. *haematobium*. For a 10 ml sample of urine, 50 *S*. *haematobium* eggs are needed for a heavy-intensity infection. For a single Kato-Katz thick smear only 41.7 mg of stool are utilized, and hence, 17 *S*. *mansoni* eggs on a slide are defined as a heavy-intensity infection. Thus, measuring an additional egg in a positive sample has a considerably larger effect in a Kato-Katz thick smear examination compared to a urine filtration test.

The age at which these morbidity indicators commonly develop was shown to hugely influence the association between infection category and microhematuria and may have an impact with other morbidity indicators. Microhematuria and ultrasound-related morbidity associated with *S*. *haematobium* has been found to be more prevalent in children, as compared to adults [48,49]. Conversely, multiple studies have shown the risk for *S*. *mansoni* morbidity indicators of periportal fibrosis to be higher in adults, as compared to children [50–52]. This could be due to *S*. *haematobium* ultrasound indicators being observable sooner than *S*. *mansoni* ultrasound indicators and with the availability of more practical ultrasound examination equipment [53], potential challenges such as this will need to be addressed if such a tool will be programmatically useful. Regardless, the likelihood of *S*. *mansoni* morbidity indicators in these school-age participants was much lower than the likelihood of *S*. *haematobium* indicators and likely contributed to the poorer associations between *S*. *mansoni*-related morbidity indicators and *S*. *mansoni* infection prevalence. In addition, the lower prevalence of *S*. *mansoni* morbidity indicators potentially created a floor effect where *S*. *mansoni* indicators had less chance to decline, as compared to, *S*. *haematobium* indicators. We conjecture that with the large sample size and analyses of ratios, we have guarded against this. Exploring how age and sex moderate relationships between morbidity and intensity categories will require a thorough evaluation.

Differences could also be due to variation in the effect of preventive chemotherapy. The follow-up time may not have been long enough for reductions in ultrasound to present at an aggregate level, especially for *S*. *mansoni*. For instance, past research suggested urinary tract pathology appears to reverse more rapidly than hepatic pathology [25]. More recent studies have suggested that *S*. *haematobium*-related morbidity improves in less than a year in school-age children [54] and in 1–2 years in adults [55], whereas liver pathologies have been shown to reduce in severity a year after initiation of treatment [56], but full resolution requires longer, approximately 2 years [57,58].

Bias or error in measurement was also possible for morbidity evaluations, though it is unclear how this affected the differences in results by species. Both *S*. *haematobium* and *S*. *mansoni* ultrasounds have had reliable inter-observer agreement [59,60], though there have been some differences in portal branch measurements [59] and portable ultrasonographic devices may have reduced sensitivity to detect bladder wall abnormalities [60]. The reliability of reagent strips is good for detecting infection [61], and a meta-analysis found that sensitivity and specificity were 81% and 89%, respectively, compared to measurement of eggs in urine [62].

In addition, some morbidities presented here are not specific to schistosome infection. For ultrasonic measures, there is the possibility for other causes of morbidity. Indeed, hepatosplenic disease in the absence of periportal fibrosis in children has been observed [63,64] and associated with current or recent malaria infection [64,65]. This could be due to some organ enlargement being caused by malaria infection [63]. There is potential that hepatitis C virus infection could lead to portal vein dilation [59], though this hypothesis is based on correlations at an aggregate level and may be due to different populations possessing different susceptibilities to liver disease [66]. Hematuria, painful urination, blood in stool, diarrhea, and abdominal pain are not solely caused by schistosomiasis and may introduce bias into the results. Self-reported morbidity also has moderate diagnostic performance [67] and many sources of bias are possible from questionnaires [68]. For these morbidities, reductions, or lack thereof, may not be wholly attributable to the decreases in infection and heavy-intensity infection observed after initiation of preventive chemotherapy.

## Conclusion

*Schistosoma* infection intensity categories are utilized to evaluate disease burden and associate with other outcomes. The current analyses found that the infection intensity categories correlated reasonably well for *S*. *haematobium* but not for *S*. *mansoni*. Control programs and researchers that utilize *Schistosoma* infection intensity categories based on egg count thresholds should be aware that the *S*. *mansoni* categories do not appear to align with the morbidity indicators used in this study and that, for both schistosome species, low-intensity infections are not morbidity-free. These analyses show that heavy-intensity infections do not capture all morbidity in these school-age children. Control program infection thresholds for *S*. *mansoni* that utilize these intensity categories based on Kato-Katz thick smear examinations should be reconfigured in order to align with morbidity levels.

## Supporting information

S1 Text Supporting information including all additional tables and figures. (DOCX)
