# Supplementary material for: Associations between infection intensity categories and morbidity prevalence in school-age children are much stronger for Schistosoma haematobium than for S. mansoni
Source: PLoS Negl Trop Dis. 2021 May 25;15(5):e0009444. doi: 10.1371/journal.pntd.0009444 (PMC8183985; doi:10.1371/journal.pntd.0009444)
Supplement: S1 Text — (DOCX) [file pntd.0009444.s001.docx]

Supplemental materials for “Associations between infection intensity categories and morbidity prevalence in children are much stronger for *Schistosoma* *haematobium* than for *Schistosoma mansoni*”.

Ryan E. Wiegand^1,2,3*^, W. Evan Secor^1^, Fiona M. Fleming^4^, Michael D. French^5^, Charles H. King^6^_,_ Arminder K. Deol^7^, Susan P. Montgomery^1^, Darin Evans^8^, Jürg Utzinger^2,3^, Penelope Vounatsou^2,3^, Sake J. de Vlas^9^

1. Division of Parasitic Diseases and Malaria, Centers for Disease Control and Prevention, Atlanta, GA, United States of America
2. Swiss Tropical and Public Health Institute, Basel, Switzerland
3. University of Basel, Basel, Switzerland
4. SCI Foundation, London, United Kingdom
5. RTI International, Washington DC, United States of America
6. Center for Global Health and Diseases, Case Western Reserve University, Cleveland, Ohio, United States of America
7. London School of Hygiene and Tropical Medicine, London, United Kingdom
8. United States Agency for International Development, Washington, DC, United States of America
9. Department of Public Health, Erasmus MC, University Medical Center Rotterdam, Rotterdam, The Netherlands

Contents

[Statistical Methods 4](#_Toc71199481)

[Details 4](#_Toc71199482)

[Implementation 6](#_Toc71199483)

[JAGS model code for *S. haematobium* logistic model 7](#_Toc71199484)

[JAGS model code for *S. mansoni* logistic model 9](#_Toc71199485)

[Supplemental Figures 11](#_Toc71199486)

[Figure A. Line graphs of the percentage of 6-15 year-old school children who were *Schistosoma haematobium*-related morbidity positive at each survey year (baseline, BL; follow-up 1, FU1; follow-up2, FU2). Estimates are from Mali, Niger, and Tanzania for all indicators. Data from Burkina Faso are included in laboratory and self-report indicators. Clustering by school accounted for in 95% confidence bands. 11](#_Toc71199487)

[Figure B. Line graphs of *Schistosoma haematobium*-related morbidity percentages including those for individual morbidity sub-scores not included in Figure 2 across three surveys (baseline, BL; follow-up 1, FU1; follow-up2, FU2). Participants were enrolled between 2003-2008 in Mali, Niger, and Tanzania. Clustering by school accounted for in 95% confidence bands. Infections were assessed by urine filtration. 12](#_Toc71199488)

[Figure C. Line graphs of the percentage of 6-15 year-old school children who were *Schistosoma mansoni*-related morbidity positive at each survey year (baseline, BL; follow-up 1, FU1; follow-up2, FU2). Estimates are from Mali, Niger, Tanzania, and Uganda. Clustering by school accounted for in 95% confidence bands. 13](#_Toc71199489)

[Figure D. Line graphs of *Schistosoma mansoni*-related morbidity percentages including morbidity scores that were not included in Figure 3, across three surveys (baseline, BL; follow-up 1, FU1; follow-up2, FU2). Participants were enrolled between 2003-2008 in Mali, Niger, Tanzania, Zambia, and Uganda. Clustering by school accounted for in 95% confidence bands. Infections were assessed by Kato-Katz. 14](#_Toc71199490)

[Figure E. Line graphs of enlarged portal vein percentage by intensity category by country across three surveys (baseline, BL; follow-up 1, FU1; follow-up2, FU2). Participants were enrolled between 2003-2008. Clustering by school accounted for in 95% confidence bands. Infections were assessed by Kato-Katz. 15](#_Toc71199491)

[Figure F. Modification of Figure 4 where participants’ *Schistosoma mansoni* intensity at each survey is split into three categories: zero epg, > 0 to < 100 epg, and ≥ 100 epg. See Figure 5 description for more details. Data are from three surveys (baseline, BL; follow-up 1, FU1; follow-up2, FU2). 16](#_Toc71199492)

[Figure G. Modification of Figure 4 where participants’ *Schistosoma mansoni* intensity at each survey is split into three categories: zero epg, > 0 to < 300 epg, and ≥ 300 epg. See Figure 5 description for more details. Data are from three surveys (baseline, BL; follow-up 1, FU1; follow-up2, FU2). 17](#_Toc71199493)

[Figure H. Modification of Figure 4 where participants’ *Schistosoma mansoni* intensity at each survey is split into three categories: zero epg, > 0 to < 400 epg, and ≥ 400 epg. See Figure 5 description for more details. Data are from three surveys (baseline, BL; follow-up 1, FU1; follow-up2, FU2). 18](#_Toc71199494)

[Supplemental Tables 19](#_Toc71199495)

[Table A. Dates of participant ascertainment in monitoring and evaluation cohorts. Shading indicates surveys that were included in analyses. Treatment was usually immediately following survey times, but occasionally lagged by a few months. 19](#_Toc71199496)

[Table B. Participants included in analyses of *Schistosoma haematobium* infection intensity categories. Numbers in parentheses indicate the number of schools from which participants were sampled. 20](#_Toc71199497)

[Table C. Odds ratios and 95% confidence intervals from Bayesian logistic regression models comparing morbidity positive proportions between *S. haematobium* intensity categories that were not included in Table 2. Bold font indicates the 95% credible interval does not contain one. Participants are school-aged children, aged 6-15 years, enrolled between 2003-2008 in Mali, Niger, and Tanzania for all indicators and Burkina Faso, Mali, Niger, and Tanzania for laboratory and self-report indicators. *S. haematobium* assessed by urine filtration. 22](#_Toc71199498)

[Table D. Participants included in cross-sectional analyses of *Schistosoma mansoni* infection intensity categories. Numbers in parentheses indicate the number of schools from which participants were sampled. 24](#_Toc71199499)

[Table E. Odds ratios and 95% confidence intervals from Bayesian logistic regression models comparing morbidity positive proportions between intensity categories within surveys for *S. mansoni*-related morbidities that were not included in Table 3. Bold font indicates the 95% credible interval does not contain one. Participants are school-aged children, aged 6-15 years, enrolled between 2003-2008 in Mali, Niger, Tanzania, and Uganda. 25](#_Toc71199500)

[Bibliography 26](#_Toc71199501)

# Statistical Methods

## Details

Our outcome in all models was the presence of a morbidity, $y_{i}$, where

$y_{i}=\left\{ \begin{aligned} 0, \text{if negative} \\ 1, \text{if positive} \end{aligned} \right.$.

We assume

$$y_{i}\sim\text{Bernoulli}(p_{i})$$

where $i$ denotes an observation. $p_{i}$ is forced to be bounded on $\left[ 0,1 \right]$. For the logistic model, we assume a logit transform of the linear predictor, specifically

$$p_{i}\text{=}\frac{e^{\eta_{i}}}{{1+e}^{\eta_{i}}}$$

where $\eta_{i}$is the linear predictor. The form of $\eta_{i}$ depends on whether participant was sampled in multiple waves. We had $n$ observations in the dataset, where ${i=1,...,n}_{1}$ come from children who were only sampled once during these three surveys and ${i=n_{1}+1,..., n}_{2}$ come from children who were only sampled more than once. For the *Schistosoma haematobium* model, with ${i=1,...,n}_{1}$ , we used the following equations since there are three intensity categories

$$\eta_{i}= \beta_{0}+\beta_{1}*\text{Light}_{i}+\beta_{2}*\text{Heavy}_{i}+ \beta_{3}*\text{FU1}_{i}+\beta_{4}*\text{FU2}_{i} +\beta_{5}*\text{Light}_{i}*\text{FU1}_{i}+\beta_{6}*\text{Light}_{i}*\text{FU2}_{i}+ \beta_{7}*\text{Heavy}_{i}*\text{FU1}_{i}+\beta_{8}*\text{Heavy}_{i}*\text{FU2}_{i}+\beta_{9_{1}}*\text{age}_{1,i}+\cdots+ \beta_{9_{j}}*\text{age}_{j,i}+\beta_{10}*\text{female}_{i}+\beta_{{11}_{1}}*\text{country}_{1,i}+\cdots+ \beta_{{11}_{j}}*\text{country}_{j,i}+\gamma_{1,k}$$

and for ${i=n_{1}+1,..., n}_{2}$ ,

$$\eta_{i}= \beta_{0}+\beta_{1}*\text{Light}_{i}+\beta_{2}*\text{Heavy}_{i}+ \beta_{3}*\text{FU1}_{i}+\beta_{4}*\text{FU2}_{i} +\beta_{5}*\text{Light}_{i}*\text{FU1}_{i}+\beta_{6}*\text{Light}_{i}*\text{FU2}_{i}+ \beta_{7}*\text{Heavy}_{i}*\text{FU1}_{i}+\beta_{8}*\text{Heavy}_{i}*\text{FU2}_{i}+\beta_{9_{1}}*\text{age}_{1,i}+\cdots+ \beta_{9_{j}}*\text{age}_{j,i}+\beta_{10}*\text{female}_{i}+\beta_{{11}_{1}}*\text{country}_{1,i}+\cdots+ \beta_{{11}_{j}}*\text{country}_{j,i}+\gamma_{1,k}+\gamma_{2,l}$$

For the *S. mansoni* model, there is the additional intensity category, meaning the model becomes

$$\eta_{i}= \beta_{0}+\beta_{1}*\text{Light}_{i}+\beta_{2}*\text{Moderate}_{i}+ \beta_{3}*\text{Heavy}_{i}+\beta_{4}*\text{FU1}_{i} +\beta_{5}*\text{FU2}_{i}+\beta_{6}*\text{Light}_{i}*\text{FU1}_{i}+ \beta_{7}*\text{Moderate}_{i}*\text{FU1}_{i}+\beta_{8}*\text{Heavy}_{i}*\text{FU1}_{i}+\beta_{9}*\text{Light}_{i}*\text{FU2}_{i}+ \beta_{10}*\text{Moderate}_{i}*\text{FU2}_{i}+\beta_{11}*\text{Heavy}_{i}*\text{FU2}_{i}+\beta_{{12}_{1}}*\text{age}_{1,i}+\cdots+ \beta_{{12}_{j}}*\text{age}_{j,i}+\beta_{13}*\text{female}_{i}+\beta_{{14}_{1}}*\text{country}_{1,i}+\cdots+ \beta_{{14}_{j}}*\text{country}_{j,i}+\gamma_{1,k}$$

and for ${i=n_{1}+1,..., n}_{2}$ ,

$$\eta_{i}= \beta_{0}+\beta_{1}*\text{Light}_{i}+\beta_{2}*\text{Moderate}_{i}+ \beta_{3}*\text{Heavy}_{i}+\beta_{4}*\text{FU1}_{i} +\beta_{5}*\text{FU2}_{i}+\beta_{6}*\text{Light}_{i}*\text{FU1}_{i}+ \beta_{7}*\text{Moderate}_{i}*\text{FU1}_{i}+\beta_{8}*\text{Heavy}_{i}*\text{FU1}_{i}+\beta_{9}*\text{Light}_{i}*\text{FU2}_{i}+ \beta_{10}*\text{Moderate}_{i}*\text{FU2}_{i}+\beta_{11}*\text{Heavy}_{i}*\text{FU2}_{i}+\beta_{{12}_{1}}*\text{age}_{1,i}+\cdots+ \beta_{{12}_{j}}*\text{age}_{j,i}+\beta_{13}*\text{female}_{i}+\beta_{{14}_{1}}*\text{country}_{1,i}+\cdots+ \beta_{{14}_{j}}*\text{country}_{j,i}+\gamma_{1,k}+\gamma_{2,l}$$

In these equations,

- $\beta$’s are the coefficient estimates (on the log odds scale),
- $\text{Light}_{i}$ is an indicator variable denoting whether observation $i$ has a light infection,
- $\text{Moderate}_{i}$is an indicator variable denoting whether observation $i$ has a moderate infection,
- $\text{Heavy}_{i}$is an indicator variable denoting whether observation $i$ has a heavy infection,
- $\text{FU1}_{i}$equals 1 when observation $i$ was ascertained in follow up 1,
- $\text{FU2}_{i}$equals 1 when observation $i$ was ascertained in follow up 2,
- $\text{age}_{i}$ is an indicator variable for the age of the participant treated as a category with 6 as the reference category to ages 7 to 15,
- $\text{female}_{i}$ is an indicator variable for whether participant is a female with male as the reference category,
- $\text{country}_{i}$ is an indicator variable for the country of the observation with $j$ countries in each model where $j$ depends on the morbidity studied, and
- $\gamma_{1,k}$ and $\gamma_{2,l}$ are random effects for school and person, respectively, where an observation is from school $k$ and person $l$, with the latter only for ${i=n_{1}+1,..., n}_{2}$.

All $\beta$’s have Cauchy prior distribution with a center of zero and a scale of 2.5.[1] For the random effect for school, $\gamma_{1,k}\sim\text{Normal}\left( 0,\tau_{1} \right)$ where $\tau_{1}={\rho_{1}}^{-2}$ and $\rho_{1}$ is given a scaled Gamma prior with 1 degree of freedom and a scale of 25. This is equivalent to the standard deviation being distributed as a half-t distribution.[2] The random effect for person follows similarly.

Binomial models are fit in the same way, except $y_{i}\sim B(t_{i},p_{i})$ where $y_{i}$ is the number of morbidity indicators present and $t_{i}$is the total number of morbidity indicator tests completed for observation $i$.

Specific estimates for the intensity categories by survey are then estimated by contrast statements.

## Implementation

Models were fit via Markov Chain Monte Carlo using JAGS[3] and CODA[4] in R via the rjags package.[5] Three chains were fit with an adaptive phase of 20,000 iterations per chain. For the final model, the iterations from the adaptive phase were discarded and each chain was run for another 100,000 iterations. After completing the 100,000 iterations, graphical displays of the trace and densities functions were used to determine if any chains or a subset of iterations should be discarded. Results were then summarized.

## JAGS model code for *S. haematobium* logistic model

model {

for (i in 1:n.1) {

y.1[i] ~ dbin(p.bound.1[i], 1)

p.bound.1[i] <- max(0, min(1, p.1[i]))

logit(p.1[i]) <- y.hat.1[i]

y.hat.1[i] <- fixed.1[i] + random2.1[i]

random2.1[i] <- r2.b0[r2.cluster.i[i]]

fixed.1[i] <- inprod(b[1:P], X.1[i,1:P])

}

for (i in 1:n.2) {

y.2[i] ~ dbin(p.bound.2[i], 1)

p.bound.2[i] <- max(0, min(1, p.2[i]))

logit(p.2[i]) <- y.hat.2[i]

y.hat.2[i] <- fixed.2[i] + random1.2[i] + random2.2[i]

random1.2[i] <- r1.b0[r1.cluster.i[i]]

random2.2[i] <- r2.b0[r2.cluster.i[i]]

fixed.2[i] <- inprod(b[1:P], X.2[i,1:P])

}

for (j in 1:r1.cluster.n) {

r1.b0[j] ~ dnorm(r1.b0.mu, r1.b0.tau)

r1.b0.hat[j] <- r1.b0.mu

}

r1.b0.mu <- 0

r1.b0.tau <- pow(r1.b0.noise, -2)

r1.b0.noise ~ dscaled.gamma(25, 1)

for (j in 1:r2.cluster.n) {

r2.b0[j] ~ dnorm(r2.b0.mu, r2.b0.tau)

r2.b0.hat[j] <- r2.b0.mu

}

r2.b0.mu <- 0

r2.b0.tau <- pow(r2.b0.noise, -2)

r2.b0.noise ~ dscaled.gamma(25, 1)

for (f in 1:P) {

b[f] ~ dt(0, pow(2.5,-2), 1)

}

c[1] <- b[4]

c[2] <- b[4] + b[6]

c[3] <- b[4] + b[7]

c[4] <- b[5]

c[5] <- b[5] + b[8]

c[6] <- b[5] + b[9]

c[7] <- b[5] - b[4]

c[8] <- b[5] + b[8] - b[4] - b[6]

c[9] <- b[5] + b[9] - b[4] - b[7]

c[10] <- b[2]

c[11] <- b[3]

c[12] <- b[3] - b[2]

c[13] <- b[2] + b[6]

c[14] <- b[3] + b[7]

c[15] <- b[3] + b[7] - b[2] - b[6]

c[16] <- b[2] + b[8]

c[17] <- b[3] + b[9]

c[18] <- b[3] + b[9] - b[2] - b[8]

for (g in 1:P) { pr.b[g] <- step(b[g]) }

for (h in 1:18) { pr.c[h] <- step(c[h]) }

}

## JAGS model code for *S. mansoni* logistic model

model {

for (i in 1:n.1) {

y.1[i] ~ dbin(p.bound.1[i], 1)

p.bound.1[i] <- max(0, min(1, p.1[i]))

logit(p.1[i]) <- y.hat.1[i]

y.hat.1[i] <- fixed.1[i] + random2.1[i]

random2.1[i] <- r2.b0[r2.cluster.i[i]]

fixed.1[i] <- inprod(b[1:P], X.1[i,1:P])

}

for (i in 1:n.2) {

y.2[i] ~ dbin(p.bound.2[i], 1)

p.bound.2[i] <- max(0, min(1, p.2[i]))

logit(p.2[i]) <- y.hat.2[i]

y.hat.2[i] <- fixed.2[i] + random1.2[i] + random2.2[i]

random1.2[i] <- r1.b0[r1.cluster.i[i]]

random2.2[i] <- r2.b0[r2.cluster.i[i]]

fixed.2[i] <- inprod(b[1:P], X.2[i,1:P])

}

for (j in 1:r1.cluster.n) {

r1.b0[j] ~ dnorm(r1.b0.mu, r1.b0.tau)

r1.b0.hat[j] <- r1.b0.mu

}

r1.b0.mu <- 0

r1.b0.tau <- pow(r1.b0.noise, -2)

r1.b0.noise ~ dscaled.gamma(25, 1)

for (j in 1:r2.cluster.n) {

r2.b0[j] ~ dnorm(r2.b0.mu, r2.b0.tau)

r2.b0.hat[j] <- r2.b0.mu

}

r2.b0.mu <- 0

r2.b0.tau <- pow(r2.b0.noise, -2)

r2.b0.noise ~ dscaled.gamma(25, 1)

for (f in 1:P) {

b[f] ~ dt(0, pow(2.5,-2), 1)

}

c[1] <- b[5]

c[2] <- b[5] + b[7]

c[3] <- b[5] + b[8]

c[4] <- b[5] + b[9]

c[5] <- b[6]

c[6] <- b[6] + b[10]

c[7] <- b[6] + b[11]

c[8] <- b[6] + b[12]

c[9] <- b[6] - b[5]

c[10] <- b[6] + b[10] - b[5] - b[7]

c[11] <- b[6] + b[11] - b[5] - b[8]

c[12] <- b[6] + b[12] - b[5] - b[9]

c[13] <- b[2]

c[14] <- b[3]

c[15] <- b[4]

c[16] <- b[3] - b[2]

c[17] <- b[4] - b[2]

c[18] <- b[4] - b[3]

c[19] <- b[2] + b[7]

c[20] <- b[3] + b[8]

c[21] <- b[4] + b[9]

c[22] <- b[3] + b[8] - b[2] - b[7]

c[23] <- b[4] + b[9] - b[2] - b[7]

c[24] <- b[4] + b[9] - b[3] - b[8]

c[25] <- b[2] + b[10]

c[26] <- b[3] + b[11]

c[27] <- b[4] + b[12]

c[28] <- b[3] + b[11] - b[2] - b[10]

c[29] <- b[4] + b[12] - b[2] - b[10]

c[30] <- b[4] + b[12] - b[3] - b[11]

for (g in 1:P) { pr.b[g] <- step(b[g]) }

for (h in 1:30) { pr.c[h] <- step(c[h]) }

}

# Supplemental Figures

## Figure A. Line graphs of the percentage of 6-15 year-old school children who were *Schistosoma haematobium*-related morbidity positive at each survey year (baseline, BL; follow-up 1, FU1; follow-up2, FU2). Estimates are from Mali, Niger, and Tanzania for all indicators. Data from Burkina Faso are included in laboratory and self-report indicators. Clustering by school accounted for in 95% confidence bands.


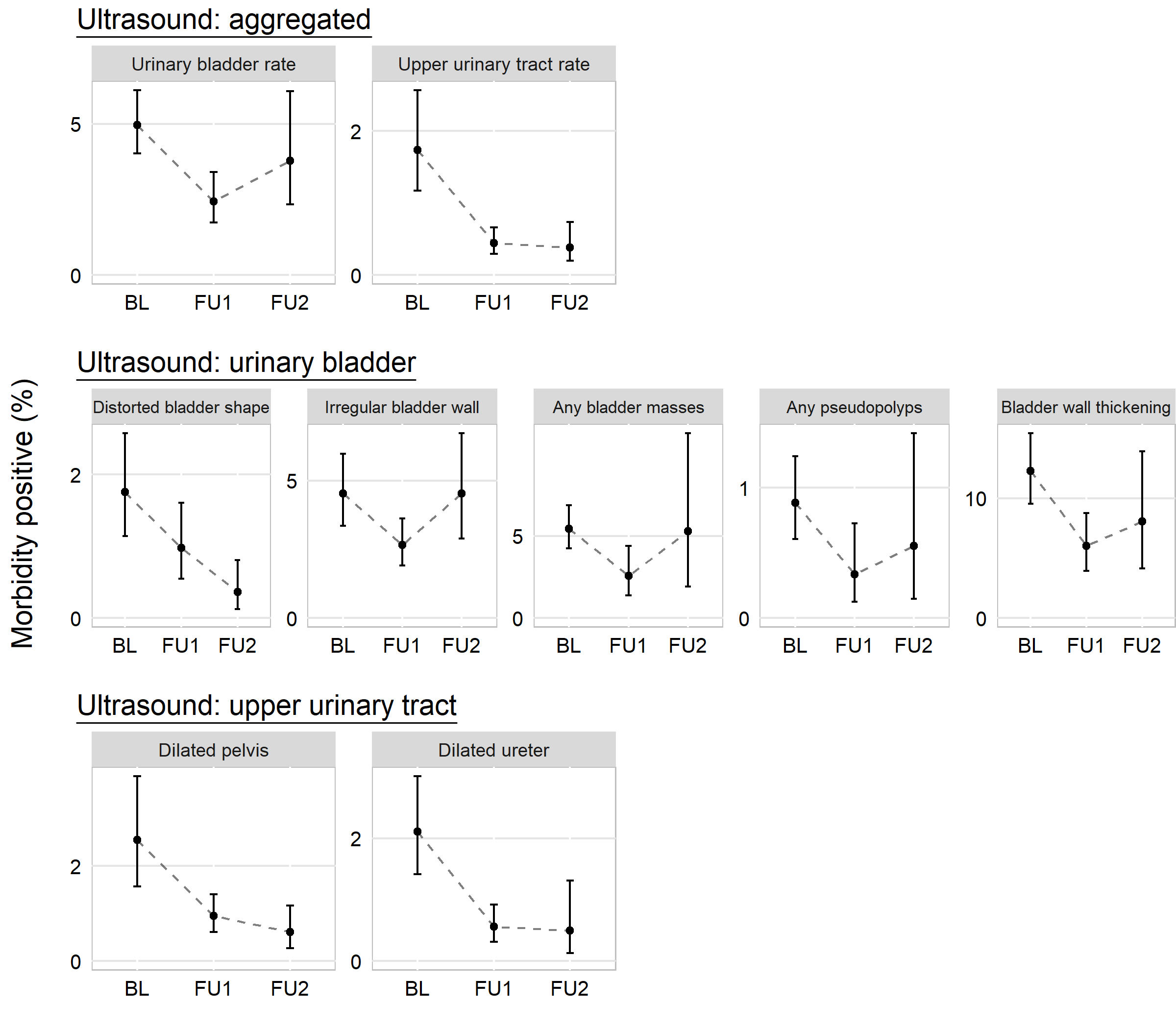


Note: Aggregated ultrasound measures include both dilated left and right pelvis and both visualized left and right ureters, but results shown for dilated pelvis and visualized ureter combine the left and right indicators into a single, binary variable of present in either or not present in either.

## Figure B. Line graphs of *Schistosoma haematobium*-related morbidity percentages including those for individual morbidity sub-scores not included in Figure 2 across three surveys (baseline, BL; follow-up 1, FU1; follow-up2, FU2). Participants were enrolled between 2003-2008 in Mali, Niger, and Tanzania. Clustering by school accounted for in 95% confidence bands. Infections were assessed by urine filtration.


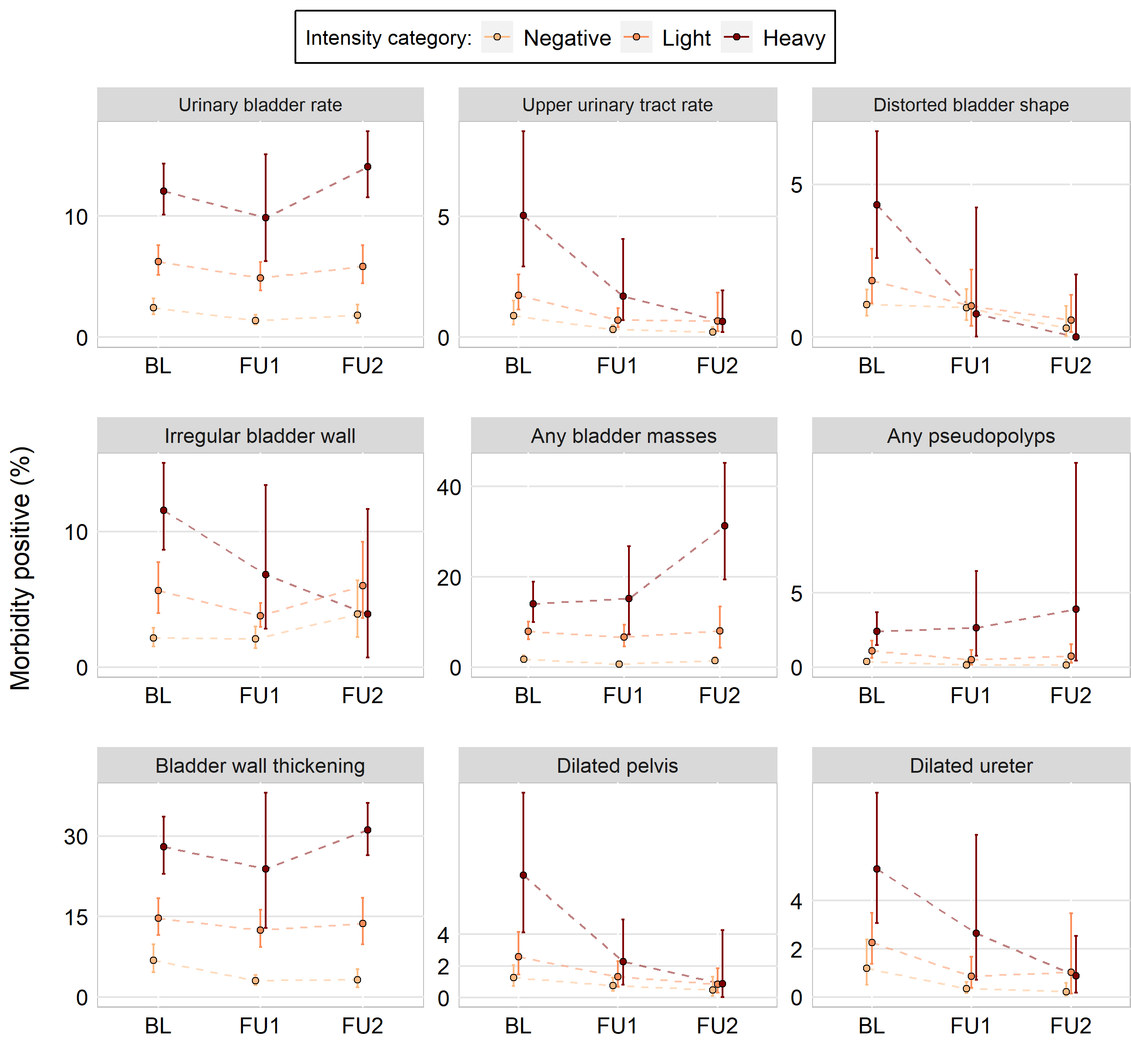


Note: Aggregated ultrasound measures include both dilated left and right pelvis and both visualized left and right ureters, but results shown for dilated pelvis and visualized ureter combine the left and right indicators into a single, binary variable of present in either or not present in either.

## Figure C. Line graphs of the percentage of 6-15 year-old school children who were *Schistosoma mansoni*-related morbidity positive at each survey year (baseline, BL; follow-up 1, FU1; follow-up2, FU2). Estimates are from Mali, Niger, Tanzania, and Uganda. Clustering by school accounted for in 95% confidence bands.


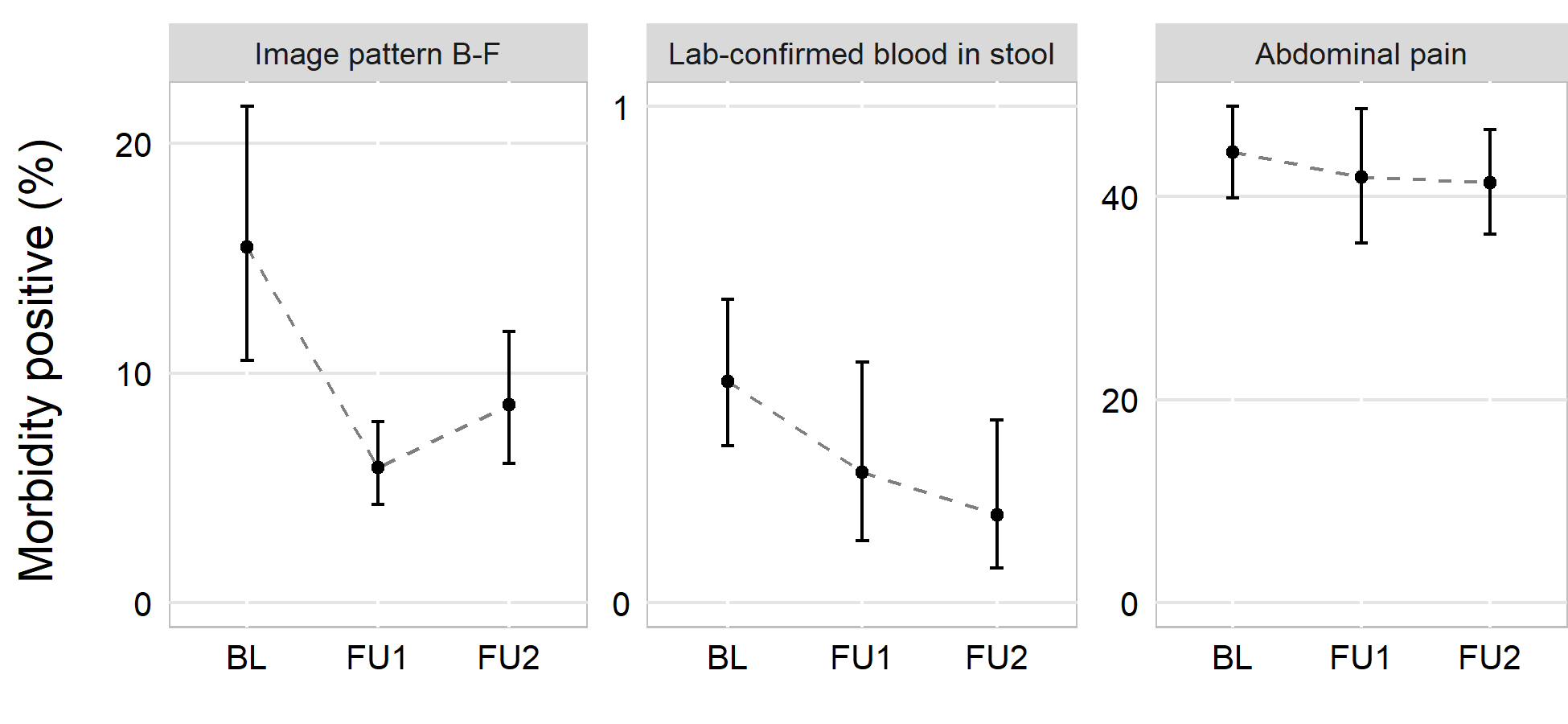


## Figure D. Line graphs of *Schistosoma mansoni*-related morbidity percentages including morbidity scores that were not included in Figure 3, across three surveys (baseline, BL; follow-up 1, FU1; follow-up2, FU2). Participants were enrolled between 2003-2008 in Mali, Niger, Tanzania, Zambia, and Uganda. Clustering by school accounted for in 95% confidence bands. Infections were assessed by Kato-Katz.


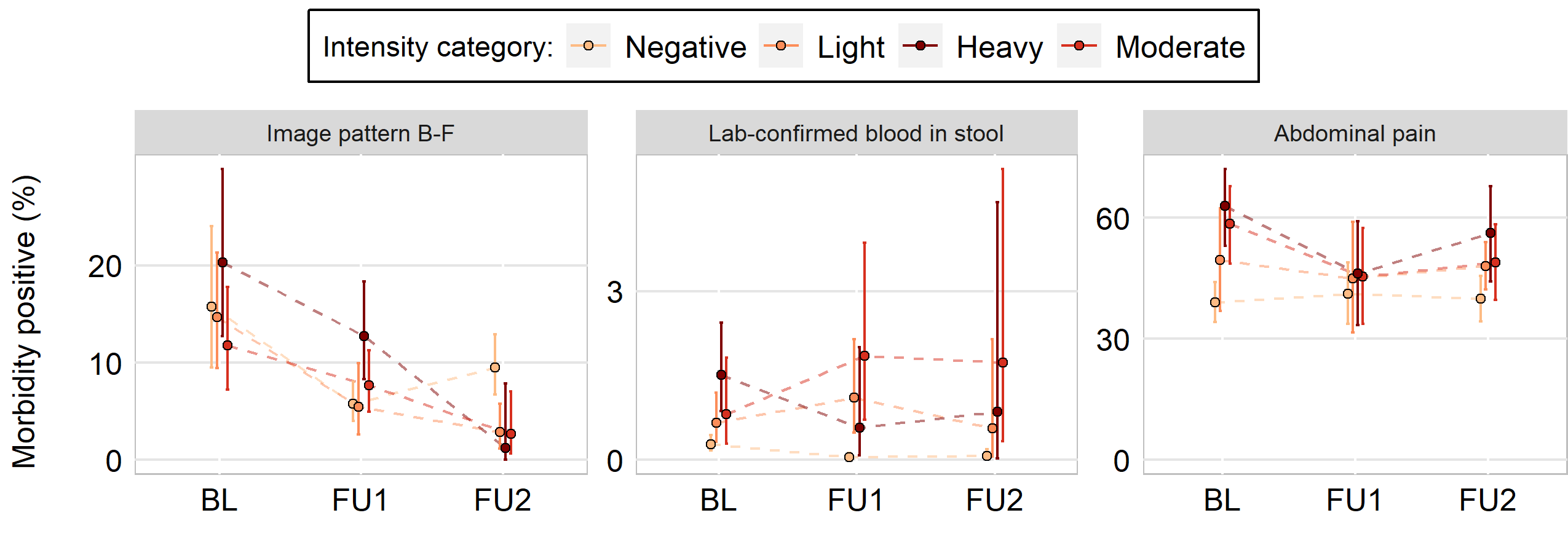


## Figure E. Line graphs of enlarged portal vein percentage by intensity category by country across three surveys (baseline, BL; follow-up 1, FU1; follow-up2, FU2). Participants were enrolled between 2003-2008. Clustering by school accounted for in 95% confidence bands. Infections were assessed by Kato-Katz.


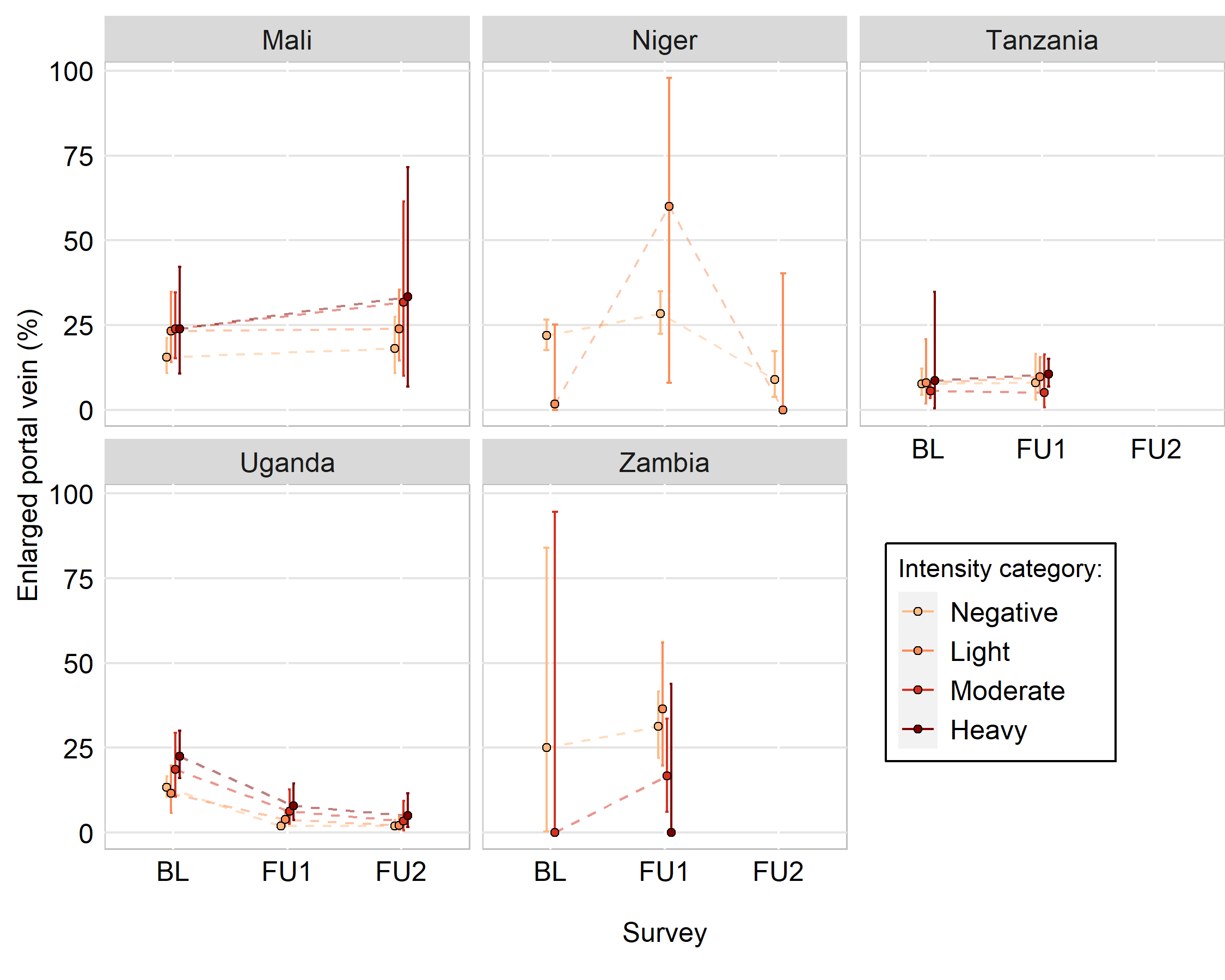


## Figure F. Modification of Figure 4 where participants’ *Schistosoma mansoni* intensity at each survey is split into three categories: zero epg, > 0 to < 100 epg, and ≥ 100 epg. See Figure 5 description for more details. Data are from three surveys (baseline, BL; follow-up 1, FU1; follow-up2, FU2).


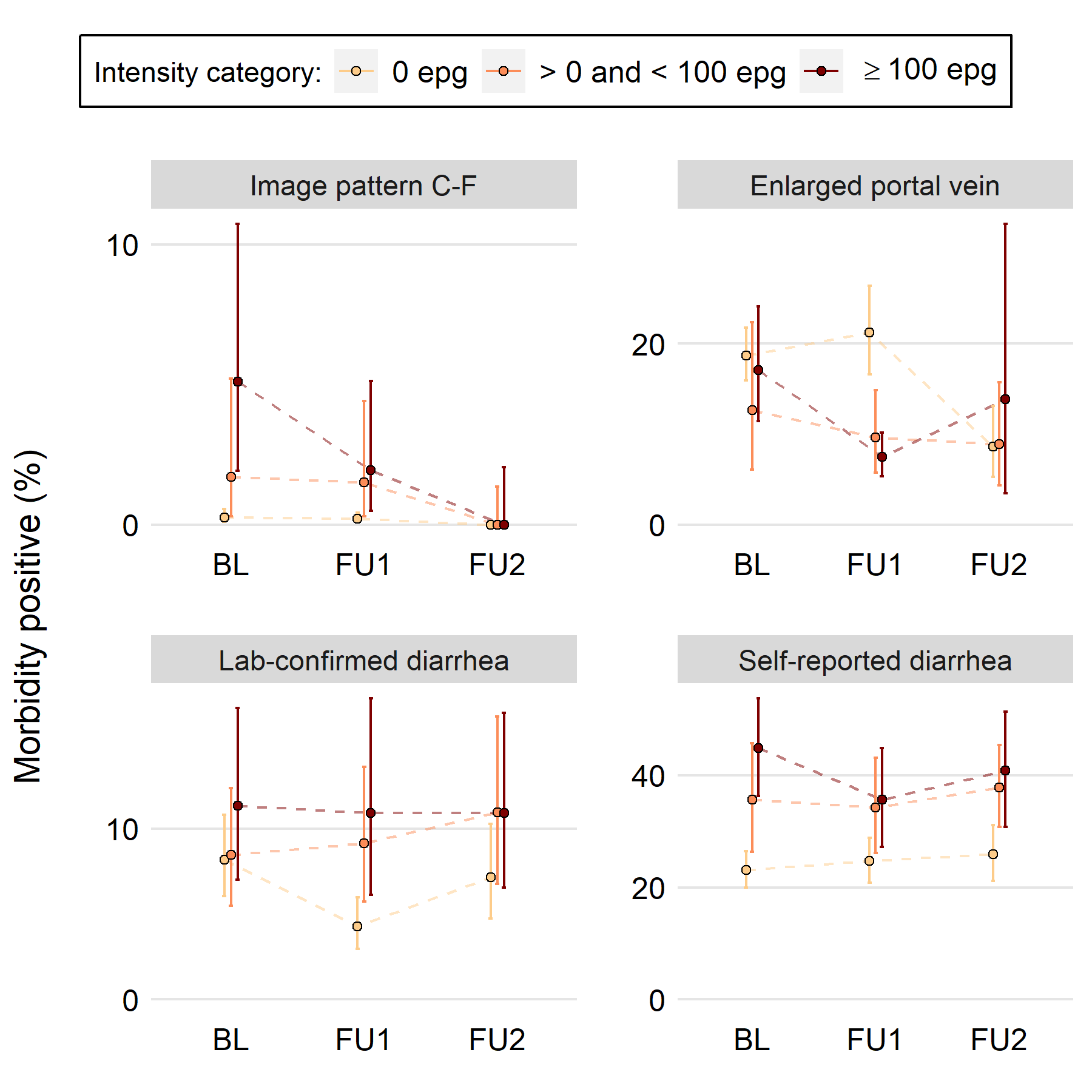


## Figure G. Modification of Figure 4 where participants’ *Schistosoma mansoni* intensity at each survey is split into three categories: zero epg, > 0 to < 300 epg, and ≥ 300 epg. See Figure 5 description for more details. Data are from three surveys (baseline, BL; follow-up 1, FU1; follow-up2, FU2).


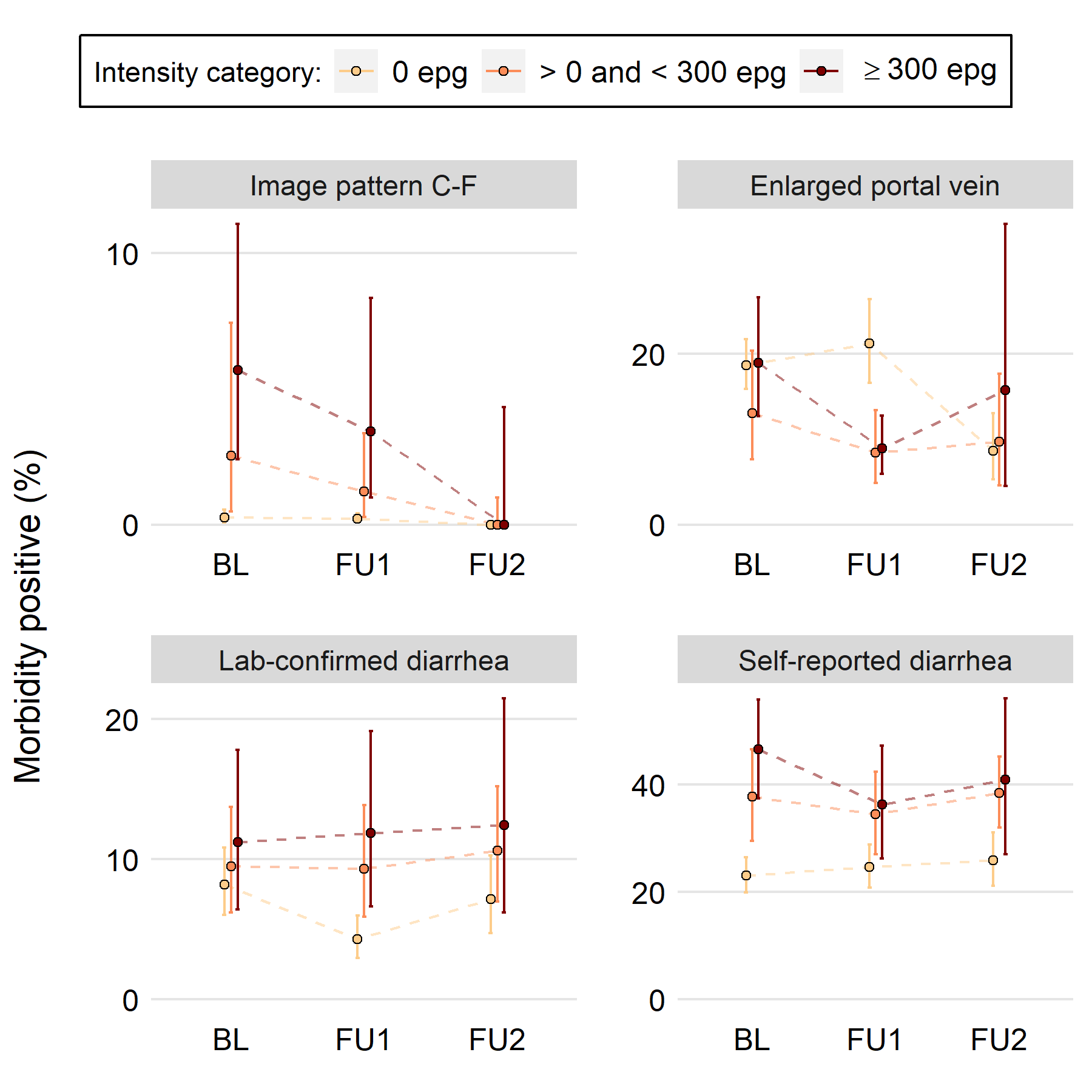


## Figure H. Modification of Figure 4 where participants’ *Schistosoma mansoni* intensity at each survey is split into three categories: zero epg, > 0 to < 400 epg, and ≥ 400 epg. See Figure 5 description for more details. Data are from three surveys (baseline, BL; follow-up 1, FU1; follow-up2, FU2).


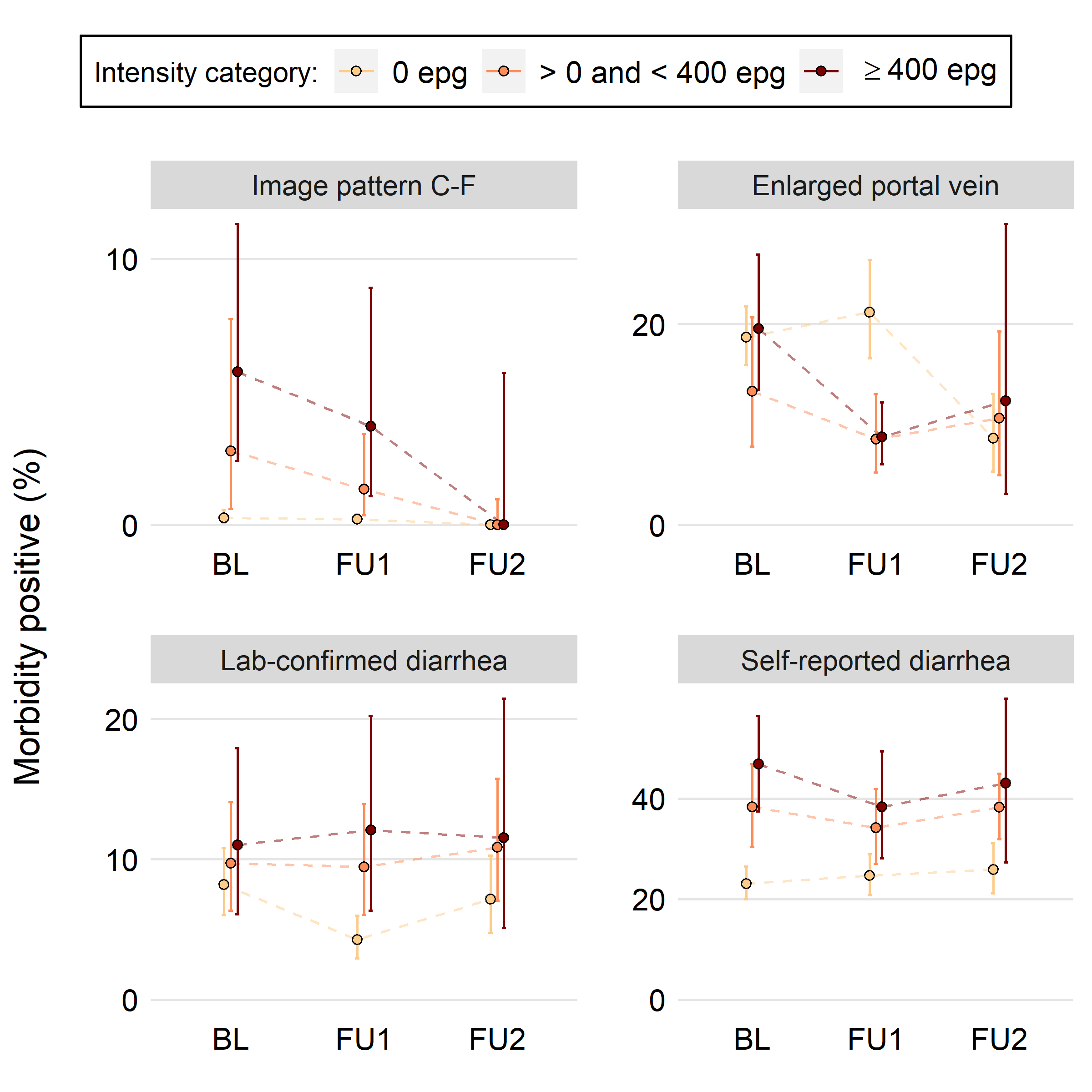


# Supplemental Tables

## Table A. Dates of participant ascertainment in monitoring and evaluation cohorts. Shading indicates surveys that were included in analyses. Treatment was usually immediately following survey times, but occasionally lagged by a few months.

| Country | Site selection | Baseline | Follow up 1 | Follow up 2 |
| --- | --- | --- | --- | --- |
| Burkina Faso | Highly endemic areas | 2004 | No treatment and all schools removed from analyses | All schools removed at prior follow up |
| Mali | Highly endemic areas | March-April 2004 (Ségou); May-July 2004 (Bamako); June-August 2004 (Koulikoro) | Two treatments during the year were performed in Ségou and those locations were dropped from further analyses; Other schools treated but not evaluated | April-May and October 2006 (Bamako); May 2006 (Koulikoro) |
| Niger | Endemic areas (5 schools); Integrated control areas with lymphatic filariasis and trachoma (3 schools) | October-November 2004 and March-May 2005 (5 schools); 2008 (3 schools) | October-December 2005 and March-April 2006 (5 schools); 2009 (3 schools) | November-December 2006 and January, March, April, and May 2007 (5 schools); February 2010 (3 schools) |
| Tanzania | Coastal areas with high prevalence | March-September 2005 | March-September 2006 | No treatment and no evaluation |
| Uganda | Eight districts with different transmission settings | February-March, March-April, October November 2003 | February-March, March-April, October-November 2004 | February-March, March-April, October-November 2005 |
| Zambia | Areas at high risk of infection | July-August 2005 and May-June 2006 | September 2006 and May-June 2007 | No treatment and no evaluation |

## Table B. Participants included in analyses of *Schistosoma haematobium* infection intensity categories. Numbers in parentheses indicate the number of schools from which participants were sampled.

|  | | Intensity category | | |
| --- | --- | --- | --- | --- |
| Morbidity | Survey | Negative | Light | Heavy |
|  |  |  |  |  |
| *Ultrasound: aggregated* |  |  |  |  |
| Any urinary bladder | Baseline | 5333 (70) | 3126 (68) | 1305 (60) |
| lesions | Follow up 1 | 4635 (37) | 1374 (34) | 264 (29) |
|  | Follow up 2 | 2313 (22) | 1058 (22) | 228 (17) |
| Any upper urinary tract | Baseline | 5305 (69) | 3118 (68) | 1315 (60) |
| lesions | Follow up 1 | 4626 (37) | 1374 (34) | 264 (29) |
|  | Follow up 2 | 2316 (22) | 1070 (22) | 229 (18) |
| Urinary bladder rate | Baseline | 5348 (70) | 3139 (68) | 1330 (60) |
|  | Follow up 1 | 4643 (37) | 1376 (34) | 264 (29) |
|  | Follow up 2 | 2324 (22) | 1071 (22) | 231 (18) |
| Upper urinary tract rate | Baseline | 5337 (69) | 3138 (68) | 1329 (60) |
|  | Follow up 1 | 4642 (37) | 1377 (34) | 264 (29) |
|  | Follow up 2 | 2325 (22) | 1072 (22) | 231 (18) |
|  |  |  |  |  |
| *Ultrasound: urinary bladder* | |  |  |  |
| Distorted bladder shape | Baseline | 5338 (70) | 3134 (68) | 1317 (60) |
|  | Follow up 1 | 4638 (37) | 1374 (34) | 264 (29) |
|  | Follow up 2 | 2317 (22) | 1063 (22) | 229 (17) |
| Irregular bladder wall | Baseline | 5343 (70) | 3138 (68) | 1323 (60) |
|  | Follow up 1 | 4643 (37) | 1376 (34) | 264 (29) |
|  | Follow up 2 | 2320 (22) | 1069 (22) | 231 (18) |
| Any bladder masses | Baseline | 5347 (70) | 3138 (68) | 1329 (60) |
|  | Follow up 1 | 4642 (37) | 1376 (34) | 264 (29) |
|  | Follow up 2 | 2320 (22) | 1069 (22) | 230 (18) |
| Any pseudopolyps | Baseline | 5346 (70) | 3135 (68) | 1325 (60) |
|  | Follow up 1 | 4641 (37) | 1376 (34) | 264 (29) |
|  | Follow up 2 | 2320 (22) | 1071 (22) | 231 (18) |
| Bladder wall thickening | Baseline | 5345 (70) | 3135 (68) | 1323 (60) |
|  | Follow up 1 | 4643 (37) | 1376 (34) | 264 (29) |
|  | Follow up 2 | 2320 (22) | 1070 (22) | 231 (18) |
|  |  |  |  |  |
| *Ultrasound: upper urinary tract* | |  |  |  |
| Dilated left or right | Baseline | 5312 (69) | 3122 (68) | 1324 (60) |
| pelvis | Follow up 1 | 4628 (37) | 1375 (34) | 264 (29) |
|  | Follow up 2 | 2318 (22) | 1071 (22) | 231 (18) |
| Visualized left or right | Baseline | 5330 (69) | 3134 (68) | 1319 (60) |
| ureter | Follow up 1 | 4639 (37) | 1376 (34) | 264 (29) |
|  | Follow up 2 | 2320 (22) | 1071 (22) | 229 (18) |
|  |  |  |  |  |
| *Laboratory and self-report* | |  |  |  |
| Microhematuria | Baseline | 6221 (91) | 3773 (88) | 1954 (77) |
|  | Follow up 1 | 5285 (41) | 1386 (36) | 270 (31) |
|  | Follow up 2 | 2354 (22) | 1084 (22) | 234 (18) |
| Pain while urinating | Baseline | 5973 (83) | 3382 (80) | 1703 (70) |
|  | Follow up 1 | 5258 (41) | 1385 (36) | 271 (31) |
|  | Follow up 2 | 2328 (22) | 1076 (22) | 233 (18) |

## Table C. Odds ratios and 95% credible intervals from Bayesian logistic regression models comparing morbidity positive proportions between *S. haematobium* intensity categories that were not included in Table 2. Bold font indicates the 95% credible interval does not contain one. Participants are school-aged children, aged 6-15 years, enrolled between 2003-2008 in Mali, Niger, and Tanzania for all indicators and Burkina Faso, Mali, Niger, and Tanzania for laboratory and self-report indicators. *S. haematobium* assessed by urine filtration.

| Morbidity | Survey | Light v. Negative | Heavy v. Negative | Heavy v. Light |
| --- | --- | --- | --- | --- |
|  |  |  |  |  |
| *Ultrasound: aggregated* |  |  |  |  |
| Urinary bladder rate | Baseline | **1.45 (1.29, 1.63)** | **1.65 (1.42, 1.90)** | 1.14 (1.00, 1.30) |
|  | Follow up 1 | **1.50 (1.31, 1.71)** | **1.42 (1.09, 1.83)** | 0.95 (0.73, 1.23) |
|  | Follow up 2 | **1.94 (1.59, 2.37)** | **2.59 (1.95, 3.42)** | **1.34 (1.01, 1.75)** |
| Upper urinary tract rate | Baseline | **1.32 (1.03, 1.69)** | **1.57 (1.18, 2.10)** | 1.19 (0.93, 1.52) |
|  | Follow up 1 | **1.45 (1.04, 2.00)** | 1.31 (0.74, 2.24) | 0.91 (0.50, 1.58) |
|  | Follow up 2 | **2.09 (1.14, 3.87)** | 1.53 (0.50, 3.98) | 0.73 (0.24, 1.87) |
|  |  |  |  |  |
| *Ultrasound: urinary bladder* | |  |  |  |
| Distorted bladder shape | Baseline | **1.91 (1.28, 2.83)** | **2.39 (1.43, 3.94)** | 1.25 (0.77, 2.01) |
|  | Follow up 1 | 1.61 (0.78, 3.10) | 2.59 (0.97, 5.89) | 1.61 (0.54, 4.44) |
|  | Follow up 2 | 0.28 (0.02, 1.39) | 1.43 (0.11, 7.29) | 5.08 (0.29, 96.12) |
| Irregular bladder wall | Baseline | **1.55 (1.21, 1.97)** | **1.93 (1.43, 2.58)** | 1.25 (0.95, 1.64) |
|  | Follow up 1 | **1.44 (1.04, 1.99)** | 1.58 (0.83, 2.80) | 1.09 (0.56, 2.00) |
|  | Follow up 2 | 1.49 (0.99, 2.22) | 1.27 (0.55, 2.58) | 0.85 (0.36, 1.76) |
| Any bladder masses | Baseline | **1.44 (1.13, 1.85)** | 1.28 (0.93, 1.75) | 0.89 (0.66, 1.18) |
|  | Follow up 1 | **1.83 (1.42, 2.38)** | **1.96 (1.21, 3.06)** | 1.07 (0.66, 1.66) |
|  | Follow up 2 | **2.35 (1.60, 3.46)** | **3.04 (1.80, 5.06)** | 1.29 (0.79, 2.07) |
| Any pseudopolyps | Baseline | 1.09 (0.61, 1.90) | 1.49 (0.74, 2.86) | 1.36 (0.68, 2.63) |
|  | Follow up 1 | 1.69 (0.86, 3.24) | 1.03 (0.19, 3.48) | 0.61 (0.11, 2.17) |
|  | Follow up 2 | 2.00 (0.57, 7.19) | **6.38 (1.52, 25.48)** | 3.19 (0.74, 12.63) |
| Bladder wall thickening | Baseline | **1.47 (1.23, 1.76)** | **1.88 (1.52, 2.33)** | **1.28 (1.05, 1.56)** |
|  | Follow up 1 | **1.44 (1.19, 1.75)** | 1.16 (0.78, 1.69) | 0.81 (0.54, 1.18) |
|  | Follow up 2 | **2.47 (1.84, 3.32)** | **3.54 (2.36, 5.29)** | 1.43 (0.97, 2.09) |
|  |  |  |  |  |
| *Ultrasound: upper urinary tract* | |  |  |  |
| Dilated left or right | Baseline | 1.35 (0.93, 1.95) | **1.92 (1.26, 2.90)** | 1.42 (0.98, 2.06) |
| pelvis | Follow up 1 | 1.39 (0.87, 2.18) | 1.85 (0.87, 3.61) | 1.34 (0.62, 2.67) |
|  | Follow up 2 | 1.68 (0.70, 4.07) | 0.69 (0.05, 3.34) | 0.41 (0.03, 2.05) |
| Visualized left or right | Baseline | 1.36 (0.91, 2.03) | 1.49 (0.92, 2.40) | 1.10 (0.70, 1.69) |
| ureter | Follow up 1 | 1.43 (0.85, 2.38) | 1.49 (0.56, 3.37) | 1.04 (0.39, 2.40) |
|  | Follow up 2 | 1.91 (0.77, 4.86) | 1.86 (0.43, 6.42) | 0.97 (0.22, 3.30) |
|  |  |  |  |  |
| *Laboratory and self-report* | |  |  |  |
| Pain while urinating | Baseline | **1.19 (1.07, 1.34)** | **1.58 (1.38, 1.81)** | **1.32 (1.15, 1.52)** |
|  | Follow up 1 | 1.02 (0.87, 1.20) | 0.93 (0.67, 1.27) | 0.91 (0.65, 1.27) |
|  | Follow up 2 | **1.27 (1.06, 1.54)** | 1.26 (0.90, 1.77) | 0.99 (0.70, 1.40) |

## Table D. Participants included in cross-sectional analyses of *Schistosoma mansoni* infection intensity categories. Numbers in parentheses indicate the number of schools from which participants were sampled.

| Morbidity | Survey | Negative | Light | Moderate | Heavy |
| --- | --- | --- | --- | --- | --- |
|  |  |  |  |  |  |
| *Ultrasound* |  |  |  |  |  |
| Irregular image | Baseline | 3590 (54) | 648 (39) | 433 (25) | 487 (23) |
| pattern (B-F or | Follow up 1 | 4229 (51) | 730 (37) | 325 (27) | 189 (24) |
| C-F) | Follow up 2 | 4009 (43) | 348 (29) | 150 (18) | 80 (17) |
| Enlarged portal | Baseline | 3539 (51) | 665 (37) | 456 (25) | 516 (23) |
| vein | Follow up 1 | 3820 (47) | 541 (36) | 326 (27) | 194 (24) |
|  | Follow up 2 | 4015 (43) | 349 (29) | 150 (18) | 81 (17) |
|  |  |  |  |  |  |
| *Laboratory* |  |  |  |  |  |
| Lab-confirmed | Baseline | 10324 (108) | 1373 (70) | 858 (50) | 928 (47) |
| blood in stool | Follow up 1 | 9417 (82) | 1178 (52) | 541 (38) | 348 (37) |
|  | Follow up 2 | 5390 (40) | 536 (34) | 173 (21) | 117 (20) |
| Lab-confirmed | Baseline | 8398 (96) | 1358 (67) | 857 (50) | 926 (47) |
| diarrhea | Follow up 1 | 6778 (74) | 1173 (50) | 541 (38) | 348 (37) |
|  | Follow up 2 | 2996 (35) | 584 (33) | 191 (23) | 130 (22) |
|  |  |  |  |  |  |
| *Self-report* |  |  |  |  |  |
| Self-reported | Baseline | 11242 (121) | 1603 (87) | 999 (61) | 1091 (55) |
| diarrhea | Follow up 1 | 9325 (80) | 1157 (50) | 524 (36) | 339 (35) |
|  | Follow up 2 | 6137 (57) | 700 (46) | 251 (29) | 151 (25) |
| Abdominal pain | Baseline | 6229 (80) | 1125 (62) | 648 (48) | 918 (43) |
|  | Follow up 1 | 5532 (46) | 858 (37) | 326 (27) | 232 (27) |
|  | Follow up 2 | 6131 (57) | 699 (46) | 251 (29) | 151 (25) |

## Table E. Odds ratios and 95% credible intervals from Bayesian logistic regression models comparing morbidity positive proportions between intensity categories within surveys for *S. mansoni*-related morbidities that were not included in Table 3. Bold font indicates the 95% credible interval does not contain one. Participants are school-aged children, aged 6-15 years, enrolled between 2003-2008 in Mali, Niger, Tanzania, and Uganda.

| Morbidity | Intensity category | Light v. Negative | Moderate v. Negative | Heavy v. Negative | Moderate v. Light | Heavy v. Light | Heavy v. Moderate |
| --- | --- | --- | --- | --- | --- | --- | --- |
| Image pattern B-F | **Baseline** | **1.74 (1.30, 2.31)** | **1.59 (1.11, 2.24)** | 1.17 (0.84, 1.62) | 0.91 (0.62, 1.34) | 0.67 (0.46, 0.98) | 0.74 (0.49, 1.11) |
|  | Follow up 1 | 1.19 (0.82, 1.69) | **1.93 (1.23, 2.95)** | **2.16 (1.24, 3.60)** | 1.62 (0.97, 2.70) | 1.82 (0.99, 3.25) | 1.12 (0.59, 2.08) |
|  | Follow up 2 | **0.56 (0.31, 0.94)** | **0.38 (0.12, 0.91)** | 0.32 (0.06, 1.02) | 0.67 (0.19, 1.90) | 0.57 (0.10, 2.05) | 0.85 (0.13, 4.27) |
| Lab-confirmed | Baseline | **2.46 (1.07, 5.23)** | 2.40 (0.88, 5.70) | **3.53 (1.49, 7.83)** | 0.98 (0.33, 2.72) | 1.44 (0.55, 3.74) | 1.48 (0.52, 4.44) |
| blood in stool | Follow up 1 | **3.50 (1.54, 7.50)** | **3.67 (1.30, 8.92)** | **3.91 (1.14, 10.79)** | 1.05 (0.35, 2.82) | 1.12 (0.31, 3.44) | 1.07 (0.27, 3.82) |
|  | Follow up 2 | 4.29 (0.91, 17.42) | 0.23 (0.00, 7.51) | 0.34 (0.00, 11.21) | 0.05 (0.00, 2.22) | 0.08 (0.00, 3.27) | * |
| Abdominal pain | Baseline | 1.13 (0.98, 1.32) | 1.14 (0.95, 1.38) | **1.32 (1.12, 1.55)** | 1.01 (0.81, 1.26) | 1.16 (0.95, 1.42) | 1.15 (0.92, 1.44) |
|  | Follow up 1 | 1.08 (0.91, 1.29) | 1.03 (0.80, 1.33) | 0.90 (0.67, 1.21) | 0.95 (0.72, 1.27) | 0.83 (0.60, 1.15) | 0.88 (0.61, 1.26) |
|  | Follow up 2 | 0.90 (0.75, 1.08) | 1.11 (0.83, 1.47) | 1.17 (0.82, 1.68) | 1.23 (0.89, 1.69) | 1.30 (0.89, 1.91) | 1.06 (0.68, 1.64) |

* The odds ratio for this effect was highly variable due to the prevalence at both surveys being close to zero. We chose to omit this effect due to its instability.

# Bibliography

1. Gelman A, Jakulin A, Pittau MG, Su Y-S. A weakly informative default prior distribution for logistic and other regression models. Ann Appl Stat. 2008;2(4):1360-83. doi: 10.1214/08-AOAS191.

2. Gelman A. Prior distributions for variance parameters in hierarchical models. Bayesian Anal. 2006;1(3):515-33.

3. Plummer M, editor JAGS: A program for analysis of Bayesian graphical models using Gibbs sampling. Proceedings of the 3rd international workshop on distributed statistical computing; 2003: Vienna, Austria.

4. Plummer M, Best N, Cowles K, Vines K. CODA: convergence diagnosis and output analysis for MCMC. R news. 2006;6(1):7-11.

5. Plummer M. rjags: Bayesian Graphical Models using MCMC. R package version 4-10 ed2019.
